# Heterozygosity at neutral and immune loci is not associated with neonatal mortality due to microbial infection in Antarctic fur seals

**DOI:** 10.1002/ece3.5317

**Published:** 2019-06-20

**Authors:** Vivienne Litzke, Meinolf Ottensmann, Jaume Forcada, Louise Heitzmann, Joseph Ivan Hoffman

**Affiliations:** ^1^ Department of Animal Behaviour Bielefeld University Bielefeld Germany; ^2^ British Antarctic Survey, High Cross Cambridge UK; ^3^ Institut des Sciences de l'Evolution CNRS Montpellier France

**Keywords:** Antarctic fur seal (Arctocephalus gazella), heterozygosity–fitness correlation (HFC), bacterial infection, inbreeding, pinniped

## Abstract

Numerous studies have reported correlations between the heterozygosity of genetic markers and fitness. These heterozygosity–fitness correlations (HFCs) play a central role in evolutionary and conservation biology, yet their mechanistic basis remains open to debate. For example, fitness associations have been widely reported at both neutral and functional loci, yet few studies have directly compared the two, making it difficult to gauge the relative contributions of genome‐wide inbreeding and specific functional genes to fitness. Here, we compared the effects of neutral and immune gene heterozygosity on death from bacterial infection in Antarctic fur seal (*Arctocephalus gazella*) pups. We specifically developed a panel of 13 microsatellites from expressed immune genes and genotyped these together with 48 neutral loci in 234 individuals, comprising 39 pups that were classified at necropsy as having most likely died of bacterial infection together with a five times larger matched sample of healthy surviving pups. Identity disequilibrium quantified from the neutral markers was positive and significant, indicative of variance in inbreeding within the study population. However, multilocus heterozygosity did not differ significantly between healthy and infected pups at either class of marker, and little evidence was found for fitness associations at individual loci. These results support a previous study of Antarctic fur seals that found no effects of heterozygosity at nine neutral microsatellites on neonatal survival and thereby help to refine our understanding of how HFCs vary across the life cycle. Given that nonsignificant HFCs are underreported in the literature, we also hope that our study will contribute toward a more balanced understanding of the wider importance of this phenomenon.

## 1 INTRODUCTION

A large and increasing number of studies are reporting positive correlations between heterozygosity, quantified from genetic markers such as microsatellites, and fitness. These heterozygosity–fitness correlations (HFCs) have been documented for a wealth of traits in many animal and plant species (David, 1998; Hansson & Westerberg, 2002; Coltman & Slate, 2003; Chapman, Nakagawa, Coltman, Slate, & Sheldon, 2009; Szulkin, Bierne, & David, 2010) and suggest that heterozygosity could be a major component of individual fitness. However, debate continues over the most likely underlying mechanisms (Hansson & Westerberg, 2002; Szulkin et al., 2010).

Two main mechanisms have been proposed to explain HFCs. First, early studies tended to assume that more homozygous individuals were less fit due to inbreeding depression (the “general effect model” (Hansson & Westerberg, 2002)). However, a second possibility is that, by chance, one or a few markers lie close enough to a gene under balancing selection such that linkage disequilibrium creates a pattern where heterozygosity at the gene and marker are correlated (the “local effect model” (David, 1998)). Both mechanisms have been criticized on theoretical grounds, general effects because of simulations suggesting that inbred individuals are unlikely to be common in large unstructured populations (Balloux, Amos, & Coulson, 2004) and local effects due to the fact that balancing selection is perceived to be rare (Kreitman & Di Rienzo, 2004; Bubb et al., 2006).

A related question surrounding HFCs concerns the number and types of genes that could be involved. Classical inbreeding depression is generally understood to be caused by many genome‐wide distributed loci of small effect size (Charlesworth & Charlesworth, 1999; Charlesworth & Willis, 2009). Conversely, HFCs caused by local effects are expected to involve relatively few genes of large effect size (Hansson & Westerberg, 2002). Moreover, the genes themselves could be diverse, or alternatively, they might be related mainly to immunity, with reduced parasite loads and disease leading to greater longevity, enhanced growth, behavioral dominance and attractiveness. There is some evidence to suggest that immune genes do play a disproportionate role, in at least some cases (Luikart, Pilgrim, Visty, Ezenwa, & Schwartz, 2008; Banks, Dubach, Viggers, & Lindenmayer, 2010; Assunção‐Franco, Hoffman, Harwood, & Amos, 2012; Lenz, Mueller, Trillmich, & Wolf, 2013; Bateson et al., 2016; Ferrer, García‐Navas, Sanz, & Ortego, 2016; Brambilla, Keller, Bassano, & Grossen, 2018). However, in general, it has proven difficult to disentangle the contributions of different genes because the vast majority of studies use neutral microsatellites, whereas studies using markers mined from functional genomic regions (Da Silva et al., 2009; Olano‐Marin, Mueller, & Kempenaers, 2011; Laine, Herczeg, Shikano, & Primmer, 2012; Ferrer et al., 2016) are still relatively uncommon.

A further problem with HFCs is publication bias. Two meta‐analyses found a significant tendency for weak or nonsignificant HFCs to go unreported in the scientific literature, particularly when sample sizes are small (Coltman & Slate, 2003; Chapman et al., 2009). The publication of null results should therefore be encouraged because publication bias has the potential to influence the perception of how strong and common HFCs actually are. Furthermore, there is a growing consensus that the ~10 to 15 microsatellite loci used in most HFC studies will in many cases have very little power to quantify variation in inbreeding (Balloux et al., 2004; Hoffman et al., 2014; Kardos, Allendorf, & Luikart, 2014; Miller & Coltman, 2014) although see Forstmeier, Schielzeth, Mueller, Ellegren, and Kempenaers (2012). Consequently, Chapman et al. (2009) questioned the merit of using small microsatellite panels and suggested that, regardless of the sample size of individuals, studies should aim to maximize the number of genetic markers.

The pinnipeds, comprising true seals, eared seals, and the walrus, are carnivorous aquatic mammals that are generally too large to suffer appreciable predation. Consequently, most non‐anthropogenic mortality is likely due to starvation, physical trauma, or pathogens. Most pinniped species also breed in highly predictable colonies and show strong site fidelity (Hoffman, Trathan, & Amos, 2006). In the past, these traits made them easy targets for hunting, but today they facilitate long‐term studies of known individuals. This predictability, coupled with interest in their wide range of breeding behaviors (Cassini, 2000; Fitzpatrick, Almbro, Gonzalez‐Voyer, Kolm, & Simmons, 2012; Krüger, Wolf, Jonker, Hoffman, & Trillmich, 2014), demographic responses to climate change (Forcada & Hoffman, 2014), and interactions with humans (Kovacs et al., 2012), has led to the development of parallel genetic studies of many related species (Stoffel et al., 2018).

Antarctic fur seals provide an ideal opportunity to investigate the genetic basis of HFCs in a wild, free‐ranging vertebrate population. A scaffold walkway constructed over a study colony facilitates access to the animals for the collection of genetic samples and life‐history data. Genetic and observational data collected since 1994 until the current day have uncovered several HFCs, with multilocus heterozygosity measured at nine neutral microsatellites being associated with multiple aspects of male reproductive success (Hoffman, Boyd, & Amos, 2004; Hoffman, Forcada, Trathan, & Amos, 2007a; Hoffman, Forcada, & Amos, 2010) as well as female recruitment and breeding success (Forcada & Hoffman, 2014). However, a study of over 1,000 pups found no effect of neutral marker heterozygosity on neonatal survival (Hoffman et al., 2006). Taken at face value, this may suggest that HFCs in this species are disproportionately important in adulthood. However, most of the pups in the former study died of trauma or starvation, conditions that are unlikely to be strongly influenced by an individual᾿s genotype. Conversely, a small proportion of pups are known to die from bacterial infection, which may well be related to heterozygosity, particularly of the immune system (Acevedo‐Whitehouse et al., 2005; Hawley, Sydenstricker, Kollias, & Dhondt, 2005; Lyons et al., 2009).

Here, we delve into greater detail by revisiting the question of whether heterozygosity influences early pup survival using three complimentary approaches. First, we enriched the current study for fur seal pups whose most likely cause of death was bacterial infection. Second, we increased the number of neutral microsatellites from nine to 48, which was sufficient to detect significant variation in inbreeding in a recent study (Stoffel et al., 2015). Third, we added an immunogenetic perspective by developing and genotyping a panel of functional markers residing within expressed candidate immune genes. We hypothesized that an HFC would be detected at the immune but not the neutral loci, in line with the results of similar studies (Luikart et al., 2008; Bateson et al., 2016; Brambilla et al., 2018).

## 2 METHODS

### 2.1 Sample collection

#### 2.1.1 Study site, data collection and tissue sampling

This study was conducted at Bird Island, South Georgia (54°00′S, 38°02′W), during the austral summers of 2000/2001–2013/2014 (hereafter breeding seasons are referred to by the year in which they began). The study population was located at a small cobblestone breeding beach (approximately 440 m^2^ at high tide) that was separated from adjacent breeding sites by a cliff on the east side, open sea on the west and rocky outcrops to the north and south. An elevated scaffold walkway (Doidge, Croxall, & Baker, 1984) provided safe access to the animals while minimizing disturbance.

Approximately 900 adult females were randomly selected and tagged using plastic cattle ear tags (Dalton Supplies) placed in the trailing edge of the foreflipper (Lunn & Boyd, 1993). Pups born to tagged females were captured on the day of birth, sexed, and weighed to the nearest 100 g in a nylon bag suspended from a 10 kg capacity spring scale (Pesola^®^ Model 220, Oskar Lüdi and Company). Piglet ear notching pliers were used to collect a small skin sample from the interdigital margin of the foreflipper, which was stored individually in 20% dimethyl sulphoxide (DMSO) saturated with salt (Amos & Hoelzel, 1991) at −20°C. The pups were then marked with temporary serial numbers by bleaching the fur on their backs before returning them to their mothers.

### 2.2 Necropsy protocol

Each season, twice‐daily surveys were made of pups born to tagged females from November 1st until the end of the pupping period in early January. Following a simplified protocol developed by a veterinarian specializing in pinniped pathology (Doidge et al., 1984), pups that died were examined both internally and externally to determine the most likely cause of death. Taking all necropsy observations into account, each dead pup was assigned to one of the following categories: (a) starvation, characterized by a thin or absent layer of subcutaneous blubber and a lack of milk in the stomach; (b) trauma, where pups exhibited traumatic injuries (e.g. crushed skull or ribs) and associated hematomas; (c) stillbirth: when the pup's umbilicus was still attached, suggesting that the individual was younger than 24 hours old at the time of death, a piece of lung tissue was removed and placed into water. Sinking tissue indicated that the pup had not taken a breath; (d) bacterial infection: in the absence of any obvious signs of trauma or starvation, the presence of lesions, pus, and/or liver discoloration indicated that infection was the most likely cause of death; and (e) unknown, including pups that were scavenged by birds or could not be assigned to any of the above categories.

### 2.3 Development and testing of immune microsatellites

The program MISA (Thiel, 2003) was used to identify short tandem repeats comprised of perfect di‐, tri‐, and tetranucleotide motifs with a minimum of five repeats from the Antarctic fur seal transcriptome assembly (Humble, Thorne, Forcada, & Hoffman, 2016). This resulted in the discovery of a total of 2,362 microsatellite loci, of which 1,687 (71.4%) were dinucleotides, 595 (25.2%) were trinucleotides and 80 (3.4%) were tetranucleotides (Table S1). We then filtered the dataset to include only microsatellites residing within contigs with gene ontology (GO) descriptions relating to immunity. Specifically, we screened the categories “cellular components,” “biological process,” and “molecular function” as well as the keywords for hits to the following list of search terms: “immun*,” “antigen,” “chemokine,” “t‐cell,” “MHC,” “antibody,” “histocompatibility,” “interleukin,” “leucocyte,” and “lymphocyte”. Loci with less than 100 bp of flanking sequence on either side of the repeat motif were then discarded to yield a total of 137 microsatellites, for which PCR primers were designed using Primer3Plus (Untergasser et al., 2007) with default settings except for the specification of an optimal primer melting temperature of 60°C.

A total of 96 microsatellite loci were selected for testing, comprising twenty that appeared polymorphic in silico as described by Hoffman and Nichols (2011) plus a further 76 loci that carried at least six repeat units (Table S2). Forward primer sequences of all 96 oligonucleotide primer pairs were equipped with a M13(−21)‐tail (TGTAAAACGACGGCCAGT), which enabled us to use the M13 system for fluorescent labeling of PCR products following Schuelke (2000). Each locus was tested for PCR amplification in twelve unrelated Antarctic fur seal individuals. PCRs were performed in single reactions with 5 μl TEMPase Hot Start 2× Master Mix (VWR, Darmstadt, Germany), 1 μl of genomic DNA, 0.25 µl forward primer (2 nM), 1 µl each of the reverse and M13(−21) universal primer (2 nM), and autoclaved water totaling a volume of 10 μl. Thermocycling using a TProfessional standard Thermocycler (Biometra GmbH, Göttingen, Germany) comprised an initial denaturation step at 95°C for 15 min followed by 26 cycles of 30 s denaturation at 95°C; 1 min annealing at either 60°C or 55°C and 1 min extension at 72°C, followed by eight additional cycles of 30 s at 94°C, 1 min at 53°C, and 1 min at 72°C. A final extension step was performed at 72°C for 4 min with subsequent cooling to 4°C. Amplification success was initially determined by running 1 μl of each PCR product on a standard 2% agarose gel for 20 min at 100 V. Reactions that failed to generate discernible PCR products at an annealing temperature of 60°C were then repeated with an annealing temperature of 55°C. Afterward, all of the PCR products were transferred to 96‐well detection plates, filled to a total volume of 20 µl with ROX size standard (1:300 dilution, range 35‒500 bp) and analyzed on an ABI 3730xl capillary sequencer (Applied Biosystems). Allele sizes were scored using the program GeneMarker version 2.6.2 (SoftGenetics^®^: State College).

### 2.4 Genotyping of focal individuals at neutral and immune microsatellites

We selected for further genotyping all pups born during the study period for which the most likely cause of death was determined at necropsy to be bacterial infection (*n* = 39 sampled over ten seasons) together with a five times larger matched sample of randomly selected healthy pups that survived until the end of the respective field seasons (*n* = 195). The sample of healthy individuals was matched in the sense that we endeavoured wherever possible to select these animals from the same years as infected individuals in order to maximize comparability. This was achieved in all years apart from 2003, where two individuals died of bacterial infection but high‐quality DNA could only be obtained for two healthy individuals. Consequently, six additional healthy individuals were genotyped from 2008 in order to match the total sample sizes.

Total genomic DNA was extracted from the skin samples using a standard phenol–chloroform protocol (Sambrook, Fritsch, & Maniatis, 1989) and genotyped at 13 immune microsatellites and 48 neutral microsatellites. The immune loci were genotyped using the M13 system as described above, while the neutral loci were genotyped using a Qiagen Type‐it kit as described by Stoffel et al. (2015). Briefly, the microsatellites were PCR amplified in five separate multiplexed reactions using the following PCR profile: one cycle of 5 min at 94°C; 24 cycles of 30 s at 94°C, 90 s at T_a_°C, and 30 s at 72°C; and one final cycle of 15 min at 72°C (see Table S3 for details of the loci together with mastermix‐specific annealing temperatures, T_a_). The resulting PCR products were subsequently resolved by electrophoresis on an ABI 3730xl and allele sizes were called relative to the LIZ size standard (1:150 dilution) using GeneMarker. To ensure genotype quality, all of the traces were manually inspected by V.L. and J.I.H and any incorrect calls were adjusted accordingly.

### 2.5 Genetic analyses

#### 2.5.1 Summary statistics

All of the loci were tested for deviation from Hardy–Weinberg equilibrium (HWE) using Genepop version 4.2 (Rousset, 2008). For each test, we set the dememorization number to 10,000, the number of batches to 1,000 and the number of iterations per batch to 10,000. False discovery rate (FDR) corrections (Benjamini & Hochberg, 1995) with an alpha level of 0.05 were applied to the resulting *p*‐values to account for multiple testing. Genepop was also used to calculate observed and expected heterozygosities as well as to determine the number of alleles amplified at each locus.

#### 2.5.2 Quantification of multilocus heterozygosity and inbreeding

Heterozygosity was quantified for each individual using the measure standardized multilocus heterozygosity (sMLH), which quantifies the proportion of loci that are heterozygous while weighting the contribution of each locus by the expected heterozygosity at that locus (Coltman, Pilkington, Smith, & Pemberton, 1999). This was performed for all of the markers combined, as well as separately for the neutral and immune markers, using inbreedR (Stoffel et al., 2016) within R version 3.5.1 (R Core Team, 2018). We furthermore used inbreedR to quantify the magnitude of identity disequilibrium (ID) by calculating the two‐locus heterozygosity disequilibrium, *g*_2_ (David, Pujol, Viard, Castella, & Goudet, 2007), and its 95% confidence interval through 1,000 permutations of the neutral microsatellite dataset.

### 2.6 Statistical analyses

To test for associations between microsatellite heterozygosity and death from bacterial infection, we constructed several alternative generalized linear mixed‐models (GLMMs) incorporating relevant predictor variables and quantified their relative support using AIC_c_ weights within a multimodel inference framework. All of the models had pup survival as a binary response variable (coded as 0 = alive and 1 = dead) and included year as a random effect to control for any variation in survivorship attributable to interannual variation. The following GLMMs were considered:

1. survival ~ 1 + (1|year)
2. survival ~ sMLH (all microsatellites) + (1|year)
3. survival ~ sMLH (immune microsatellites) + (1|year)
4. survival ~ sMLH (neutral microsatellites) + (1|year)
5. survival ~ 1 + birth weight + (1|year)
6. survival ~ sMLH (all microsatellites) + birth weight + (1|year)
7. survival ~ sMLH (immune microsatellites) + birth weight + (1|year)
8. survival ~ sMLH (neutral microsatellites) + birth weight + (1|year)

These included “null models” without any genetic effects (models one and five) as well as models that included sMLH combined over all loci or calculated separately for the neutral versus the immune loci. Models five to eight also included pup birth weight (in kg) to incorporate any potential effects of body size on survivorship. All of the models were specified using the glmer function of the package “lme4” (Bates, Maechler, Bolker, & Walker, 2014) with a binomial error structure. Using the R package AICcmodavg, the most parsimonious model was selected based on the delta AIC_c_ value, which compares weights as a measure of the likelihood of a particular model (Burnham & Anderson, 2004; Mazerolle, 2013). The best supported model has ∆AIC_c_ = 0 and a difference of two or more units was applied as a criterion for choosing one model over a competing model (Burnham & Anderson, 2004). Furthermore, we tested for local effects by fitting linear models again with survival as the response variable and heterozygosity (0 = homozygous, 1 = heterozygous) as a fixed effect applied for each locus individually.

## 3 RESULTS

In order to facilitate a comparison of neutral and functional loci, we developed a panel of microsatellites from expressed immune genes. Out of a total of 96 putative microsatellite loci that were tested in 12 individuals using the M13 approach, 11 produced clearly interpretable and polymorphic PCR products (Table S4). A further 60 loci yielded monomorphic PCR products while the remaining loci either failed to amplify or did not produce interpretable products.

To investigate the relative contributions of functional and neutral loci to fitness variation, we genotyped a total of 234 fur seals comprising 39 pups that died of bacterial infection together with a five times larger matched sample of healthy pups. Additionally, we tested for cross‐amplification in six grey seal and six northern elephant seal individuals. A total of 48 neutral markers were genotyped in five mastermixes as well as 13 immune microsatellites, including the 11 loci described above plus two previously published immune markers (Hoffman & Nichols, 2011). None of the microsatellites violated the assumptions of Hardy–Weinberg equilibrium in the Antarctic fur seal after table‐wide false discovery rate correction (Table S3).

**Figure 1:**
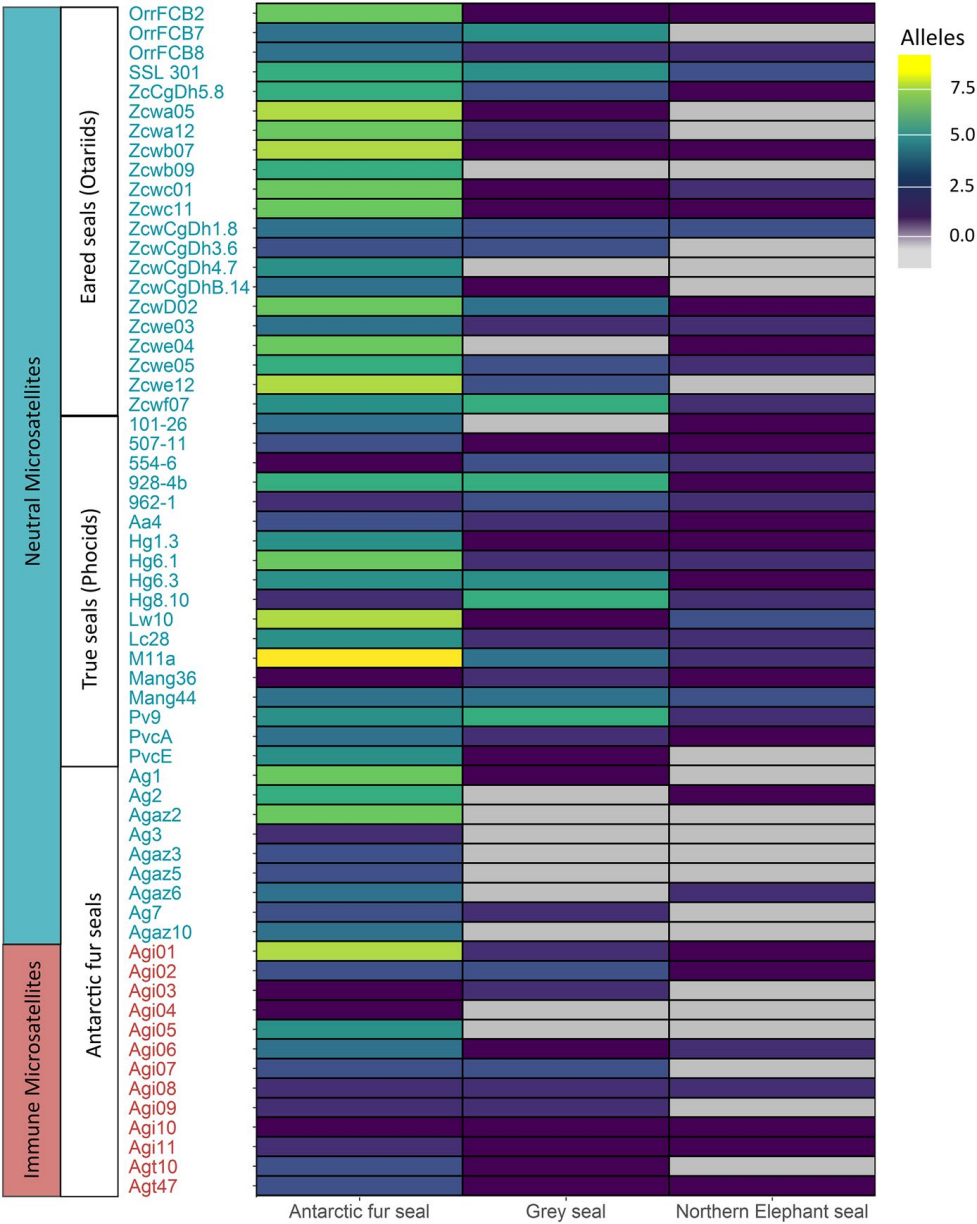
Heatmap depicting variation within and across species in the allelic richness of neutral and functional loci. To allow visual comparison across species, the number of alleles was quantified for six randomly sampled Antarctic fur seal individuals

Figure 1 reveals a number of patterns in the distribution of genetic variability among species and different types of genetic marker. Focusing initially on the Antarctic fur seals, allelic richness was higher at the neutral loci relative to the immune loci (7.52 ± 2.75 [mean, *SD*] vs. 4.62 ± 2.50 [mean, *SD*] respectively). If we assume that our estimates of variability are not biased by elements of marker development, this difference is significant (Mann–Whitney *U* test, W = 137.5, *p* = 0.002). Furthermore, the neutral markers tended to carry more alleles when they were cross‐amplified from other species as opposed to developed in Antarctic fur seals (8.13 ± 2.55 [mean, *SD*] vs. 4.89 ± 2.03 [mean, *SD*], Mann–Whitney *U* test, W = 59, *p* = 0.002). Finally, cross‐species amplification rates were significantly higher for microsatellites developed in the Antarctic fur seal from immune versus neutral genomic regions (cross‐amplification rate = 0.69 vs. 0.22 respectively, Mann–Whitney *U* test, W = 124, *p* = 0.003).

**Figure 2:**
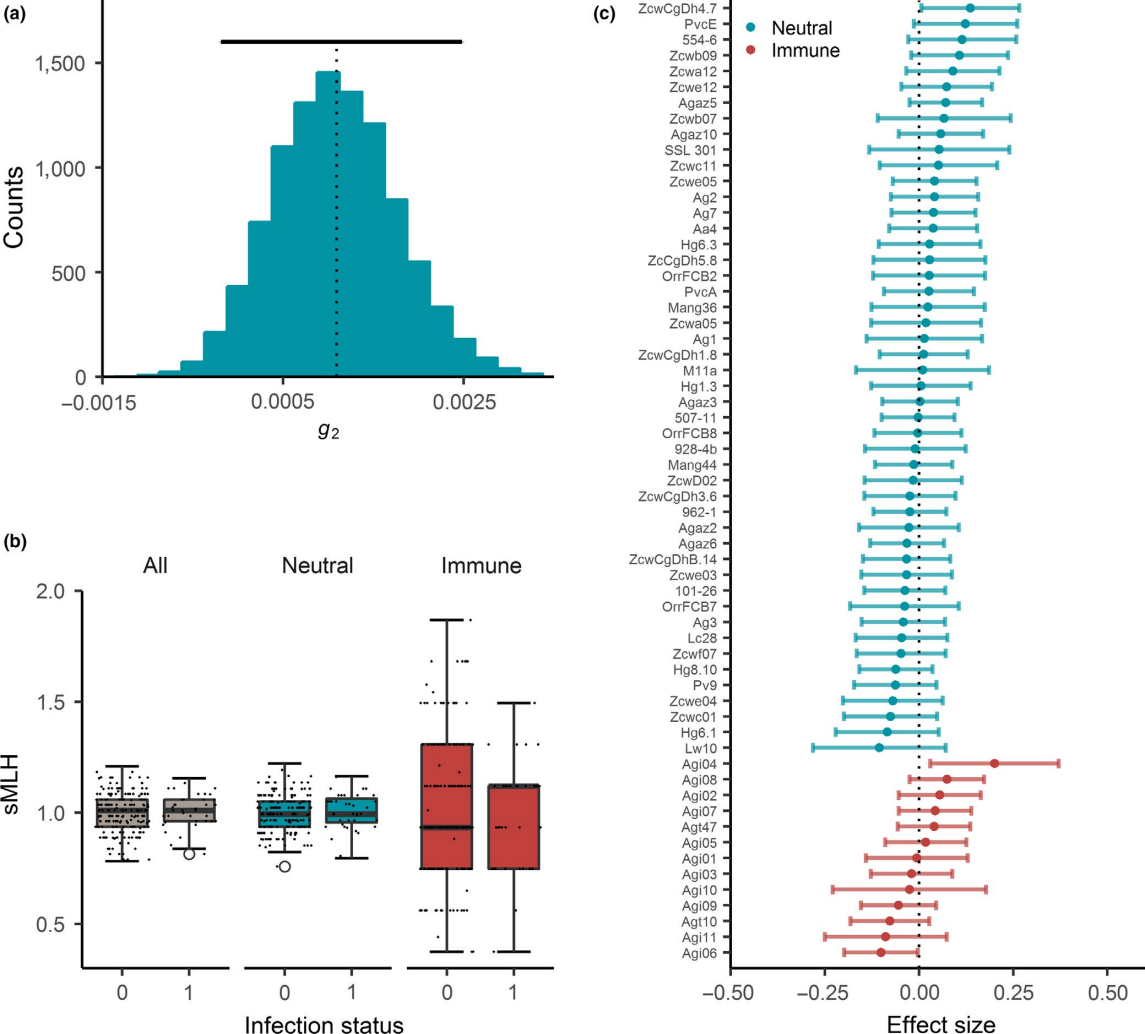
Variance in inbreeding and the effects of neutral and immune loci on death from bacterial infection in Antarctic fur seal pups. (a) Distribution of identity disequilibrium (*g*_2_) estimates obtained by bootstrapping data from 48 neutral loci genotyped in 234 individuals. The vertical dashed line represents the empirical *g*_2_ estimate, and the horizontal black line shows the corresponding 95% confidence interval based on 1,000 bootstrap replicates. (b) Variation in standardized multilocus heterozygosity (sMLH) between healthy pups that survived until the end of the breeding season and pups that died of bacterial infection (coded as 0 and 1, respectively). The raw data points are shown together with standard Tukey's box plots. (c) Effect sizes and associated 95% confidence intervals for each locus, ranked by descending order of effect size separately for neutral and immune microsatellites

To quantify the variance in inbreeding within the Antarctic fur seal population, we calculated the two‐locus disequilibrium (*g*_2_) based on the neutral loci. Our estimate (0.0011, 95% confidence interval, CI = −0.0002 to 0.0025, Figure 2a) was similar in magnitude to a previous study of the same population and was statistically significant (*p* = 0.033). To test for effects of heterozygosity on death from bacterial infection, we then used a multimodel inference approach to compare alternative models, including null models with no genetic terms as well as models containing sMLH calculated respectively for the neutral loci, the immune loci, and all of the loci combined (see Materials and methods for details). The best supported model contained only the intercept together with year fitted as a random effect, although three other models, the first containing birth weight as a fixed effect, the second containing neutral marker sMLH, and the third containing overall sMLH, were ranked within two ΔAIC_c_ units of the best model, indicating that they have a similar level of statistical support (Table 1). Model averaged effect size estimates for sMLH based on the neutral loci, the immune loci, and all of the loci combined are given in Table 2. sMLH did not differ appreciably between healthy and infected pups, regardless of which class of marker was used (Figure 2b). Furthermore, average effect sizes did not differ significantly between the neutral and immune markers (0.009 ± 0.001 [mean, *SEM*] and 0.005 ± 0.006 [mean, *SEM*], respectively, Wilcoxon rank sum test, *p* = 0.721). In addition, all but one of the 61 markers had effect sizes with 95% CIs overlapping zero (Figure 2c), suggesting that overall there is little evidence of local effects.

**Table 1:** Alternative models of pup survival ranked in order of their AIC_c_ support

Model	Structure	*k*	AIC_c_	∆AIC_c_	AIC_c_ weight	Log likelihood
1	survival ~ 1 + (1|year)	2	214.91	0.00	0.27	−105.43
5	survival ~ 1 + birth weight + (1|year)	3	215.56	0.64	0.19	−104.73
4	survival ~ sMLH (neutral microsatellites) + (1|year)	3	216.66	1.74	0.11	−105.28
2	survival ~ sMLH (all microsatellites) + (1|year)	3	216.74	1.83	0.11	−105.32
3	survival ~ sMLH (immune microsatellites) + (1|year)	3	216.97	2.05	0.10	−105.43
8	survival ~ sMLH (neutral microsatellites) + birth weight + (1|year)	4	217.43	2.51	0.08	−104.62
6	survival ~ sMLH (all microsatellites) + birth weight + (1|year)	4	217.47	2.56	0.07	−104.65
7	survival ~ sMLH (immune microsatellites) + birth weight + (1|year)	4	217.63	2.71	0.07	−104.73

**Table 2:** Model‐averaged parameter estimates for multilocus genetic terms

Term	Effect size	95% CI
sMLH neutral microsatellites	1.08	−3.07, 5.23
sMLH immune microsatellites	0.02	−1.06, 1.11
sMLH all microsatellites	0.88	−3.04, 4.81
Birth weight	−0.23	−0.81, 0.34

## 4 DISCUSSION

Heterozygosity–fitness correlations are widespread across the animal kingdom but remain poorly understood because most studies still use too few genetic markers to capture variation in inbreeding (Hansson & Westerberg, 2002; Balloux et al., 2004; Szulkin et al., 2010). We therefore deployed a large number of neutral microsatellites in combination with a newly developed panel of immune microsatellites to test for an HFC for neonatal mortality due to bacterial infection in a colonially breeding marine mammal. We found no obvious differences in either neutral or immune gene heterozygosity between healthy and infected pups. Our results therefore suggest that heterozygosity has little influence on early mortality in Antarctic fur seals, despite strong effects being found later in life.

### 4.1 Immune microsatellite development, polymorphism levels and cross‐amplification

By focusing our microsatellite development effort on immune loci residing within expressed transcripts, we generated a new marker panel that captures genetic variation across predominantly functional regions of the genome. Reassuringly, genetic variability expressed as allelic richness was lower for this immune marker panel, consistent with the notion that stronger selection on functional genomic regions constrains microsatellite evolution, regardless of whether the microsatellites themselves have direct functional effects or whether they are in linkage disequilibrium with nearby functional polymorphisms (Li, Korol, Fahima, Beiles, & Nevo, 2002; Li, Korol, Fahima, & Nevo, 2004). However, it is also possible that the immune loci may have lower allelic richness simply because the requirement for the immune markers to be in specific genomic locations restricts the choice of available sequences. We also found that cross‐species amplification rates of microsatellites developed in Antarctic fur seals were higher for immune versus neutral loci, which again can be interpreted as suggesting that selection on functional regions of the genome constrains microsatellite mutation over evolutionary timescales (Dufresnes, Brelsford, Béziers, & Perrin, 2014). However, it is also possible that the improved cross‐amplification of immune loci could be a reflection of their lower variability as microsatellite mutability tends to scale positively with allele number (Seyfert et al., 2008).

We also found that neutral microsatellites cross‐amplified from other pinniped species tended to show higher levels of variability in Antarctic fur seals than microsatellites developed in the focal species. This observation may help to explain two previous HFC studies that found stronger associations between heterozygosity and fitness using markers cloned from other species (Hoffman, Hanson, Hanson, Forcada, Trathan, & Amos, 2010; Küpper et al., 2010). One possible explanation for this difference could be that loci showing persistently high levels of polymorphism across species are better able to detect local effects because they are biased in favour of loci near genes under balancing selection (Hoffman et al., 2010). However, our findings hint at a potential alternative explanation as cross‐amplified loci may also provide greater power to detect inbreeding effects due to their higher allelic richness. Regardless of the exact explanation, our study highlights a practical difficulty in quantifying genetic variability and its fitness consequences from microsatellites, as it is a common practice to cross‐amplify loci from other species but this may result in biased parameter estimates.

### 4.2 Variance in inbreeding in Antarctic fur seals

We deployed a large panel of neutral microsatellites to test for inbreeding effects on mortality due to bacterial infection. In line with recent studies based on both microsatellites (Stoffel et al., 2015) and genomic data (Humble et al., 2018), we obtained a significantly positive *g*_2_ estimate, which is indicative of variance in inbreeding within the study population. This is to be expected given the highly polygynous breeding system of Antarctic fur seals (Hoffman, Boyd, & Amos, 2003) and their extreme site fidelity (Hoffman et al., 2006; Hoffman & Forcada, 2012). Consequently, given that multiple HFCs have already been found in this species with only nine neutral microsatellites (Hoffman et al., 2004, 2007, 2010; Forcada & Hoffman, 2014), assuming that the mechanism is inbreeding depression, our larger panel of neutral markers should have greater power to detect fitness effects (Hoffman et al., 2014).

### 4.3 Fitness effects of neutral and immune heterozygosity

We found no effect of multilocus heterozygosity on pup survival, in contrast to studies of several other pinniped species including grey seals (Bean et al., 2004), harbour seals (Rijks, Hoffman, Kuiken, Osterhaus, & Amos, 2008), and California sea lions (Acevedo‐Whitehouse et al., 2006). However, our results are in line with a previous study of Antarctic fur seals that did not find an HFC for neonatal mortality (Hoffman, Forcada, & Amos, 2006). Consequently, it seems reasonable to conclude that inbreeding is unlikely to have a major influence on neonatal survival in this species. However, the two studies are not strictly comparable as Hoffman et al. (2006) analyzed a random sample of pups that included many individuals dying from starvation or trauma, which are unlikely to be strongly influenced by genetic factors. By contrast, we focused the current study on pups whose most likely cause of death was bacterial infection, a trait that is known to correlate with heterozygosity in several species (Acevedo‐Whitehouse et al., 2005; Hawley et al., 2005; Lyons et al., 2009).

Given that bacterial infection status may have a genetic basis and is likely to be associated with variation in immune activity, we compared and contrasted fitness effects at neutral and functional genomic regions related to immunity. Previous studies of other species using a similar approach have uncovered mixed results, with some studies finding comparable HFC effect sizes at neutral and functional loci (Da Silva et al., 2009; Ferrer et al., 2016), others finding trait‐ and age‐specific differences in the strength of association between the two classes of microsatellite (Olano‐Marin et al., 2011) and at least two studies detecting HFCs at functional but not neutral loci (Bateson et al., 2016; Brambilla et al., 2018). By contrast, neither neutral nor immune microsatellites appear to have an appreciable effect on mortality due to bacterial infection in Antarctic fur seals, either when combined within classes to produce multilocus heterozygosity measures or when analyzed individually. However, we cannot discount the possibility that immune effects are present but could not be detected with the specific set of genes represented by our immune marker panel. This could be remedied in future studies by using approaches capable of interrogating the immune system more thoroughly such as whole exome capture.

### 4.4 Statistical power

Heterozygosity–fitness correlation effect sizes are often small, with multilocus heterozygosity typically explaining only around 1–5% of trait variance (Chapman et al., 2009). Consequently, there is a possibility that HFC studies obtaining null results might simply lack the statistical power to detect an effect that is actually present. More generally, it is a common practice among biologists to advocate *post hoc* power analyses as a means of interpreting tests with nonsignificant results. However, the approach of calculating the “observed power” given the value of a test statistic is flawed because there is a one to one relationship between the *p*‐value and the power to reject the null hypothesis of no effect: in other words, the higher the *p*‐value, the lower the power (Steidl, Hayes, & Schauber, 1997; Hoenig & Heisey, 2001; Levine & Ensom, 2001; Colegrave & Ruxton, 2002). As explained by Hoenig and Heisey (2001), “computing the observed power after observing the *p*‐value should cause nothing to change about our interpretation of the *p*‐value”.

As *post hoc* power calculations do not provide a meaningful way of evaluating negative results, many authors have advocated providing confidence intervals for effect sizes (Steidl et al., 1997; Hoenig & Heisey, 2001; Levine & Ensom, 2001; Colegrave & Ruxton, 2002). The upper and lower CIs are influenced by both sample size and the variance of the data, and therefore the breadth of the CI provides an indication of the likelihood of the true effect size being zero. Furthermore, the presentation of effect sizes and associated CIs facilitates direct comparisons among studies as well as meta‐analyses (Nakagawa & Cuthill, 2007). For these reasons, we have provided effect sizes and their associated 95% CIs throughout our manuscript. In general, our CIs are quite large, which implies that our effect size estimates are rather imprecise. However, this seems to be a general issue with the HFC literature and is a reflection of the difficulty of collecting large individual‐based genetic and life‐history datasets. Moreover, previous studies of Antarctic fur seals have detected HFCs for traits including recruitment success (Forcada & Hoffman, 2014) and body size (Hoffman et al., 2010) using smaller sample sizes of both individuals and loci than the current study. Consequently, if there is an effect of heterozygosity on neonatal survival in Antarctic fur seals, the current results would suggest that this is  weak in comparison to later acting effects.

A further caveat is that the term “bacterial infection” could potentially encompass one main type of bacterial pathogen or a number of different pathogens. Unfortunately, precise data on the bacterial strains present in pups that died of bacterial infection are lacking. We therefore cannot rule out the possibility that an HFC for bacterial infection could not be detected due to confounding interactions between host heterozygosity and multiple bacterial strains. Nevertheless, regardless of the exact cause of death, our results in combination with those of Hoffman et al. (2006) suggest that dead and healthy Antarctic fur seal pups do not differ appreciably in their heterozygosity.

### 4.5 Publication bias

Setting our findings into a broader context, correlations between heterozygosity and fitness have been reported in diverse organisms, suggesting that HFCs are a genuine and biologically important phenomenon. However, the field of HFCs as a whole suffers from publication bias, with around 10% of all HFC effect sizes going unreported in the literature (Chapman et al., 2009). These missing effect sizes are enriched for weak or nonsignificant HFCs originating mainly from smaller studies, an observation that led Coltman and Slate (2003) to conclude that many HFC studies may have been conducted as an afterthought using microsatellite datasets collected for other purposes. A related issue is that HFC studies tend to be underpowered to detect variation in inbreeding, as too few genetic markers are usually used to be able to detect significant identity disequilibrium (Kardos et al., 2014; Miller & Coltman, 2014). Although ours is only a single study, it was purposely designed to test for HFCs and employed a larger than usual panel of genetic markers. The outcome of our study was clear in the sense that there was little evidence for any genetic effects on fitness despite the neutral markers demonstrably capturing variation among individuals in inbreeding. We therefore concur with Chapman et al. (2009) that HFC studies should seek to increase marker numbers, especially when the sample size of individuals cannot be enlarged.

## 5 CONCLUSION

Although inbreeding occurs in our study population and can be detected with our panel of neutral markers, neither neutral nor immune microsatellite heterozygosity was associated with neonatal mortality due to bacterial infection in Antarctic fur seals. Taken together with the results of a previous study (Hoffman et al., 2006), our results therefore suggest that heterozygosity may have its strongest effects in Antarctic fur seals after weaning (Forcada & Hoffman, 2014) and in adulthood (Hoffman et al., 2004, 2007, 2010). More generally, our study contributes toward a small but growing body of research (Hoffman et al., 2006; Borrell et al., 2004; Dahle, Zedrosser, & Swenson, 2006; Taylor et al., 2010; Chapman & Sheldon, 2011; Zeng, Li, Zhang, Zhong, & Jiang, 2013; Vanpé et al., 2015; Hicks & Rachlow, 2006) reporting no associations between heterozygosity and fitness in natural populations.

## CONFLICT OF INTEREST

The authors declare no competing interests.

## AUTHORS' CONTRIBUTIONS

JIH and VL conceived the study. JF collected and transported the samples. VL and MO conducted the laboratory work under the supervision of JIH. VL and JIH scored the genotypes. MO, VL, and LH analyzed the data. JIH and VL wrote the manuscript, and all of the authors edited and approved the final manuscript.

## Data Availability

Code and documentation are available as a PDF file written in Rmarkdown (File S1). Raw data and scripts for the analysis and the development of microsatellite markers are available via Dryad (https://doi.org/10.5061/dryad.vk4br80).
