# Supplementary material for: Heterozygosity at neutral and immune loci is not associated with neonatal mortality due to microbial infection in Antarctic fur seals
Source: Ecol Evol. 2019 Jun 20;9(14):7985–96. doi: 10.1002/ece3.5317 (PMC6662382; doi:10.1002/ece3.5317)
Supplement: Supplementary file 1 [file ECE3-9-7985-s001.pdf]

R-code for 'Heterozygosity at neutral and immune loci does not influence neonatal mortality due to microbial infection in Antarctic fur seals'

Vivienne Litzke, Meinolf Ottensmann, Jaume Forcada, Louise Heitzmann & Joseph I. Hoffman

This document provides all the `R code` used in our paper. Both the Rmarkdown file and the data can be downloaded from the accompanying GitHub repository on (https://github.com/vlitzke/HeterozygosityPupSurvival) as a zip archive containing all the files. We recommend to download or clone this GitHub repository in order to access the documentation together with all the files that are needed to repeat analyses shown in this document. Just click on the link above and then on the green box `Clone or download`. In order to function properly, the same structure of folders must be kept. If you have any questions, don't hesitate to contact vivienne.litzke[at]mail.mcgill.ca

The data originates from samples collected from a colony of Antarctic fur seals (*Arctocephalus gazella*) at Bird Island, South Georgia between the years of 2000 and 2014. We investigated the effects of neutral and immune gene heterozygosity on early mortality due to bacterial infection using the `inbreedR` package.[1]

- If you have downloaded the project from github then you will see that:
- The raw data required are located in the folder `data/`
- The *Arctocephalus gazella* transcriptome[2] may be downloaded here and saved as `arc_gaz_transcriptome.fasta` in `data`.
- This pipeline invokes the `MIcroSAtellite identification tool` for primer identification. Click on the following link for details and how to install it: MISA[3].
- Primer development was conducted using primer3[4].
- Additionally, the `R packages` listed below are required and may be installed on your system.

Download packages and libraries

In order to repeat analyses presented in this manuscript a number of packages that extend the functionalities of base `R` are required. These can be installed using the code shown below.

```
install.packages('inbreedR')
install.packages("readxl")
install.packages("ggplot2")
install.packages("gridExtra")
install.packages("stringi")
install.packages("adegenet")
install.packages("AICcmodavg")
install.packages("reshape2")
install.packages("kableExtra")
```

[1] Stoffel, M. A., Esser, M., Kardos, M., Humble, E., Nichols, H., David, P., & Hoffman, J. I. (2016). inbreedR: an R package for the analysis of inbreeding based on genetic markers. Methods in Ecology and Evolution, 7(11), 1331-1339.

[2] Humble, E., Thorne, M.A., Forcada, J. & Hoffman, J.I., (2016). Transcriptomic SNP discovery for custom genotyping arrays: impacts of sequence data, SNP calling method and genotyping technology on the probability of validation success. BMC research notes, 9(1), p.418.

[3] Thiel, T., (2003). MISA—Microsatellite identification tool. Website http://pgrc. ipk-gatersleben.

[4] Untergasser, A., Nijveen, H., Rao, X., Bisseling, T., Geurts, R. & Leunissen, J.A., (2007). Primer3Plus, an enhanced web interface to Primer3. Nucleic acids research, 35(suppl_2), pp.W71-W74.

```r
source("https://bioconductor.org/biocLite.R")
biocLite("qvalue")
```

```r
library(inbreedR)
library(readxl)
library(magrittr)
library(ggplot2)
library(grid)
library(gridExtra)
library(AICcmodavg)
library(Matrix)
library(lme4)
library(qvalue)
library(adegenet)
library(reshape2)
library(kableExtra)
```

In order to use `inbreedR`, the working format is typically an *individual x loci* matrix, where rows represent individuals and every two columns represent a single locus. If an individual is heterozygous at a given locus, it is coded as 1, whereas a homozygote is coded as 0, and missing data are coded as NA.

The first step is to read the data from an excel file. Our original table includes, plate number, well number, species, id, year, health status (represented by a binomial with 0 for healthy and 1 for infected), birth weight, and the following markers (a and b for alleles).

```r
## read data
seals <-  readxl::read_xlsx("data/data.xlsx")[1:234, ]
## express alleles as numerals
seals[8:ncol(seals)] <- lapply(seals[8:ncol(seals)], as.numeric)
```

Here's an example of what the data frame looks like:

```r
head(seals[1:6,4:11])
```

```
## # A tibble: 6 x 8
##   ID     Year  `Health status` Birthweight  Agt47.a Agt47.b Agt10.a Agt10.b
##   <chr>  <chr> <chr>           <chr>          <dbl>   <dbl>   <dbl>   <dbl>
## 1 AGP00~ 2000  0               5.099999999~     237     245     213     213
## 2 AGP00~ 2000  1               4.8              241     245     213     213
## 3 AGP00~ 2000  0               4.099999999~     237     241     213     213
## 4 AGP00~ 2000  0               4.8              237     241     213     213
## 5 AGP00~ 2000  0               5.2              241     241     213     215
## 6 AGP00~ 2000  0               5.9              241     241     213     213
```

Since demographic data is present in the beginning of our data frame, we will start our new genotype file from the 8th column onwards. The function `convert_raw` converts a common format for genetic markers (two columns per locus) into the `inbreedR` working format. Afterwards, `check_data` allows us to test whether the genotype data frame has the correct format for subsequent analyses that use `inbreedR` functions.

```r
seals_geno <- convert_raw(seals[8:ncol(seals)])
invisible(lapply(seals_geno, table, useNA = "always"))
check_data(seals_geno, num_ind = 234, num_loci = 61)
```

Separate markers

Divide the neutral and immune markers from their respective columns in the adjusted inbreedR format, and compute standard multilocus heterozygosity (sMLH).[5]

```
## subset markers based on type
imm_msats <- seals_geno[, 1:13]
neut_msats <- seals_geno[, 14:61]

## estimate sMLH
all_het <- sMLH(seals_geno)
neutral_het <- sMLH(neut_msats)
immune_het <- sMLH(imm_msats)
```

Create and reshape dataframes

Take out id, health, marker types, and birth weight as variables. Tack on the year.

```
sealdf <- data.frame(id = seals[[4]],  health = factor(seals[[6]]),
                       All = all_het, Neutral = neutral_het, Immune = immune_het)

sealdf_reshaped <- reshape2::melt(sealdf)

birthweight <- as.numeric(as.character(seals[["Birthweight"]]))

sealdf_weight <- data.frame(id = seals[[4]],  health = factor(seals[[6]]), birthweight,
                             All = all_het, Neutral = neutral_het, Immune = immune_het)

sealdf_year <- cbind(sealdf_weight, year = seals[[5]])
```

Calculate g_2

g_2 is a proxy for identity disequilibrium. It is a measure of two-locus disequilibrium, which quantifies the extent to which heterozygosities are correlated across pairs of loci.[6] This allows us to take a look at our neutral marker heterozygosity to determine if there is variation in inbreeding in the population.

Plot the distribution of g2 estimates:

```
g2_neut_bs_hist <-
  ggplot2::ggplot() +
  theme_classic() +
  geom_histogram(binwidth = 0.00025, data = g2_neut_bs, aes(x = bs),
                 color = "#0294A5",
                 fill = "#0294A5") +
  geom_errorbarh(data = g2_neut_bs,
                 aes(y = 1600, x = g2, xmin = lcl, xmax = ucl),
                 color = "black", size = 0.7, linetype = "solid") +
  geom_linerange(data = g2_neut_bs,
                 aes(ymin = 0, ymax = 1600, x = g2),
```

[5] Coltman, D. W. and J. Slate. 2003. Microsatellite measures of inbreeding: a meta-analysis. Evolution 57:971–983.
[6] David, P., Pujol, B., Viard, F., Castella, V., & Goudet, J. (2007). Reliable selfing rate estimates from imperfect population genetic data. Molecular ecology, 16(12), 2474-2487.

```
                 linetype = 'dotted') +
  theme(text = element_text(size = 12),
        panel.border = element_blank(),
        strip.background =element_rect(fill = "white", colour = "white"),
        strip.text = element_text(colour = 'white'),
        plot.margin = grid::unit(c(2,2,2,2), 'mm')) +
  facet_wrap(~p) +
  ylab(" ") +
  ylab("Counts") +
  labs(x = expression(italic(g)["2"])) +
  ggtitle("a)") +
  scale_y_continuous(expand = c(0,0), limits = c(0,1800)) +
  scale_x_continuous(limits = c(-0.0015, 0.0035),
                     breaks = seq(-0.0015, 0.0035, 0.002),
                     expand = c(0,0))
```

Plot heterozygosity among marker sets

In order to visualize sMLH for all, neutral, and immune markers, create the following box-plot:

```
het_plot <-
  ggplot(data = sealdf_reshaped, aes(x=health, y=value, fill = variable)) +
  stat_boxplot(aes(x = health, y = value),
               geom='errorbar', linetype=1, width=0.5) +
  geom_boxplot( aes(x = health, y = value),outlier.shape=1) +
  geom_jitter(shape=16, position=position_jitter(0.4), size = .1) +
  theme_classic() +
  theme(legend.position = "none",
        panel.border = element_blank(),
        strip.background = element_blank(),
        text = element_text(size = 12),
        plot.margin = grid::unit(c(2,2,2,2), 'mm')) +
  xlab("Infection status") +
  ylab("sMLH") +
  ggtitle("b)") +
  scale_fill_manual(values =  c("#A79C93", "#0294A5", "#C1403D")) +
  facet_wrap(~variable, nrow = 1) +
  scale_y_continuous(limits = c(0.3, 2),
                     expand = c(0,0))
```

Estimating heterozygosity for individual loci

As we have previously looked at genome-wide effects, it may be of interest to look for local effects. Therefore, we wanted to examine the heterozygosity for each locus. First, define the confidence interval:

```
confidence_interval <- function(vector) {
  ## standard deviation
  vec_sd <- sd(vector)
  ## sample size
  n <- length(vector)
  ## sample mean
  vec_mean <- mean(vector)
  ## error according to t distribution
  error <- qt((.95 + 1)/2, df = n - 1) * vec_sd / sqrt(n)
  ## confidence interval as a vector
  result <- c("lower" = vec_mean - error, "upper" = vec_mean + error)
  return(result)
}
```

Calculate the heterozygosity for each locus, and use a regression on infection status:

```
## calcaute sMLH
het_per_locus <- apply(seals_geno, 2, sMLH)
## add factors
df <-  cbind(sealdf, seals_geno)
## add marker type as names to the data.frame
names(df)[6:66] <- c(paste0("I", 1:13), paste0("N", 1:48))


lm_by_loc <- lapply(1:61, function(x) {
  ## extract data of given marker x
  value <- df[,x + 5]
  ## run linear models
  res <- summary(lm(as.numeric(df$health) ~ value))
  conf <- confint(lm(as.numeric(df$health) ~ value))
  f <- res$fstatistic
  pf(f[1], f[2], f[3], lower=FALSE)
  out <- data.frame(beta = res$coefficients[2,1],
                    lcl = conf[2,1],
                    ucl = conf[2,2])
}) %>%
  do.call("rbind",.) %>%
  cbind(., data.frame(names = colnames(seals)[seq(8, ncol(seals), 2)] %>%
                        substring(., first = 1, last = nchar(.) - 2),
                      type = c(rep("Immune", 13),rep("Neutral", 48)),
                      dummy = ""))

## order by effect size
lm_by_loc <- lm_by_loc[with(lm_by_loc, order(type, beta, decreasing = F)),]
lm_by_loc$num <- 1:61

## create data frame to label effects
names_df <-  data.frame(label = lm_by_loc$names,
                        num = lm_by_loc$num)
```

Create a plot to feature each loci and their relevant effect sizes:

```
het_by_loci_plot <- ggplot(lm_by_loc, aes(x = num, y = beta, col = type)) +
  geom_errorbar(aes(ymin = lcl, ymax = ucl),
              width=0.6, alpha=0.7, size = 0.7) +
  geom_point(size = 1) +
  scale_x_continuous(expand = c(0,0), breaks = 1:61, labels = names_df$label) +
  scale_y_continuous(expand = c(0,0)) +
  geom_hline(yintercept = 0, linetype = "dotted") +
  coord_flip(xlim = c(0, 61.5), ylim = c(-0.5,0.6)) +
  scale_color_manual(values=c("#C1403D","#0294A5"),
                     name = "",
                     breaks = c("Neutral", "Immune"),
                     labels = c("Neutral", "Immune")) +
  theme_classic() +
  xlab("") +
  ylab("Effect size") +
  theme(legend.justification = c(0,1),
        legend.position = c(0,1.05),
        legend.background = element_rect(fill = NA),
        text = element_text(size = 12),
        axis.text.y = element_text(size = 5),
        legend.text = element_text(size = 7),
        panel.border = element_blank(),
        strip.background =element_rect(fill = "white", colour = "white"),
        strip.text = element_text(colour = 'white'),
        plot.margin = grid::unit(c(2,2,2,2), 'mm')) +
  guides(color = guide_legend(
    keywidth = 0.05,
    keyheight = 0.05,
    default.unit = "inch")) +
  facet_wrap(~dummy) +
  ggtitle("c)")
```

First we can look at the mean and standard error between neutral and immune loci, then we can look for local effects between the effect sizes of neutral and immune loci, using a Wilcoxon test:

```
mean(lm_by_loc$beta[1:13])
sd(lm_by_loc$beta[1:13])/13

mean(lm_by_loc$beta[14:61])
sd(lm_by_loc$beta[14:61])/48

wilcox.test(lm_by_loc$beta[1:13],lm_by_loc$beta[14:61])
```

Create the final combined figure

To create a combination plot of all figures (as in the manuscript):

```
## define layout of the plot
lay <- rbind(c(1,3),
             c(2,3))
```

```r
## combine figures
combo_plot <- grid.arrange(g2_neut_bs_hist,
                           het_plot,
                           het_by_loci_plot, ncol = 3, layout_matrix = lay)
```

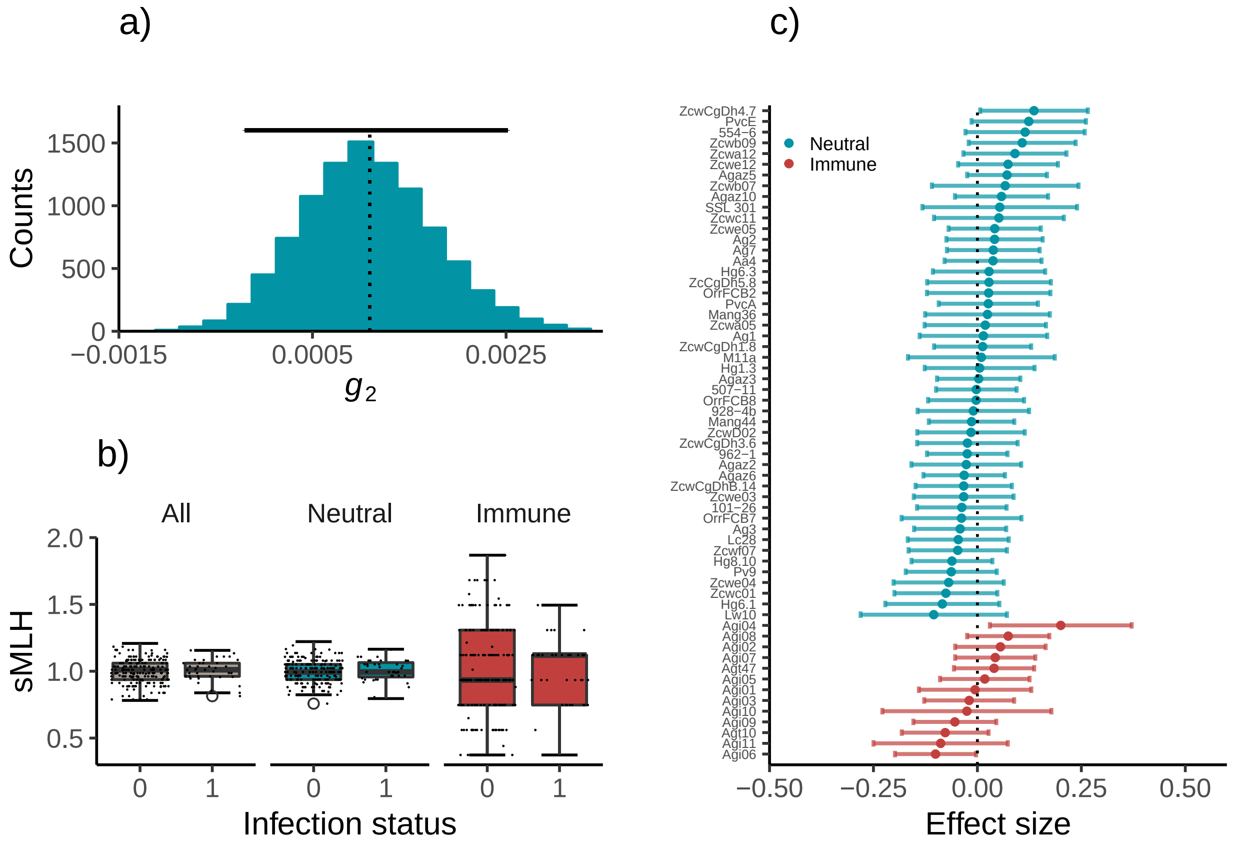

Modeling effects of sMLH on bacterial infection status

To test for associations between microsatellite heterozygosity and death from bacterial infection, we constructed several alternative generalized linear mixed-models (GLMMs) incorporating relevant predictor variables and quantified their relative support using AICc weights within a multi-model inference framework. All of the models had pup survival as a binary response variable (coded as 0 = alive and 1 = dead) and included year as a random effect to statistically control for any variation in survivorship attributable to inter-annual variation. The following GLMMs were considered:

```r
## define models
models <- list(
  glmer(health ~  1 + (1|year), data = sealdf_year, family = 'binomial'),
  glmer(health ~  All + (1|year), data = sealdf_year, family = 'binomial'),
  glmer(health ~  Immune + (1|year), data = sealdf_year, family = 'binomial'),
  glmer(health ~  Neutral + (1|year), data = sealdf_year, family = 'binomial'),
  glmer(health ~  1 + birthweight + (1|year), data = sealdf_year, family = 'binomial'),
  glmer(health ~  All + birthweight + (1|year), data = sealdf_year, family = 'binomial'),
  glmer(health ~  Immune + birthweight + (1|year), data = sealdf_year, family = 'binomial'),
  glmer(health ~  Neutral + birthweight + (1|year), data = sealdf_year, family = 'binomial'))
```

```
## boundary (singular) fit: see ?isSingular
## boundary (singular) fit: see ?isSingular
## boundary (singular) fit: see ?isSingular
## boundary (singular) fit: see ?isSingular
## boundary (singular) fit: see ?isSingular
## boundary (singular) fit: see ?isSingular
## boundary (singular) fit: see ?isSingular
## boundary (singular) fit: see ?isSingular
```

```
names(models) <- paste0("m", 1:length(models))

## model selection using delta AICc
kableExtra::kable(AICcmodavg::aictab(models, second.ord = T), booktabs = TRUE,
                  longtable = FALSE, caption = "Model selection")

# Model averaged paramaters, one by one
AICcmodavg::modavg(models, parm = "All")
AICcmodavg::modavg(models, parm = "Immune")
AICcmodavg::modavg(models, parm = "Neutral")
AICcmodavg::modavg(models, parm = "birthweight")
```

These included 'null models' without any genetic effects (models i and v) as well as models that included sMLH combined over all loci or calculated separately for the neutral versus immune loci. Models v to viii also included pup birth weight (in kg) to incorporate any potential effects of body size on survivorship. All of the models were specified using the glmer function of the package "lme4" with a binomial error structure.[7] Using the R package `AICcmodavg`, the most parsimonious model was selected based on the delta AICc value, which compares weights as a measure of the likelihood of a particular model.[8] The best supported model has Δ AICc = 0 and a difference of two or more units was applied as a criterion for choosing one model over a competing model.[9]

Supplementary Data

(a) Patterns of allelic richness and cross-amplification

We tested for patterns in allelic richness among markers (i.e. immune vs neutral), developmental source (i.e. designed for Antarctic fur seals, phocids or otariids). Secondly, we evaluate the cross-amplification success of loci in two other species of pinnipeds, namely the Grey seal and Nothern Elephant seal.

```
## read and format genotypes
library(readxl)
heatmap_df <- readxl::read_xlsx("data/data.xlsx")[, c(3, 8:ncol(seals))]

## randomly select six individuals per species
heatmap_df <- heatmap_df[c(sample(which(heatmap_df[["Species"]] == "Fur seal"),
                                   size = 6, replace = F),
           sample(which(heatmap_df[["Species"]] == "Grey seal"),
                  size = 6, replace = F),
           sample(which(heatmap_df[["Species"]] == "Northern Elephant seal"),
```

[7]Bates, D., Maechler, AICcmodavg::aictab(models, second.ord = T)M., Bolker, B., & Walker, S. (2014). Fitting linear mixed-effects models using lme4. arXiv preprint arXiv:1406.5823.

[8]Mazerolle, M. J., & Mazerolle, M. M. J. (2017). Package 'AICcmodavg'. R package.

[9]Anderson, D. R., & Burnham, K. P. (2002). Avoiding pitfalls when using information-theoretic methods. The Journal of Wildlife Management, 912-918.

```r
                  size = 6, replace = F)),]

## extract geno
marker_geno <- apply(heatmap_df[,-1], 2, as.character)

## get loci names
loci_names <- colnames(marker_geno)[seq(1, ncol(marker_geno), 2)] %>%
  substring(., first = 1, last = nchar(.) - 2)

## define a vector of immune marker names
immune_marker_names <- c("Agi01", "Agi02", "Agi03", "Agi04",
                         "Agi05", "Agi06", "Agi07", "Agi08",
                         "Agi09", "Agi10", "Agi11", "Agt10", "Agt47")

## collapse information for each locus in one column
marker_geno <- lapply(seq(1, ncol(marker_geno), 2), function(x) {
  marker_geno[,x:(x + 1)] %>%
    apply(., 1, paste0, collapse = "/")
}) %>%
  do.call("cbind",.) %>%
  ## rename loci
  set_colnames(x = ., value = paste0("Locus", 1:61))

## set missing data to NA
marker_geno[which(marker_geno == "NA/NA")] <- NA

## convert to GENIND object
genind <- adegenet::df2genind(marker_geno, ploidy = 2, sep = "/",
                              pop = heatmap_df[["Species"]] %>% as.factor)

## convert to GENPOP
genpop <- adegenet::genind2genpop(genind)
```

```r
heatmap_df <- lapply(levels(genpop@loc.fac), function(i) {
  df.temp <- genpop@tab[,which(genpop@loc.fac == i)]   ## fetch data
  if (is.null(dim(df.temp))) {
    df.temp[df.temp > 0] <- 1
    df.temp[df.temp == 0] <- 0

  } else {
    df.temp <-   apply(df.temp, 2, function(x) ifelse(x > 0, 1, 0)) %>%
      ## presence/absence of allele
    rowSums(na.rm = T) ## count alleles
  }
  # return results
  return(data.frame(Species = names(df.temp),
                    Locus = i,
                    Alleles = df.temp))
}) %>%
  do.call("rbind", .)

## set zero to NA
heatmap_df[["Alleles"]][which(heatmap_df[["Alleles"]] == 0)] <- NA
```

```r
heatmap_df[["Locus"]] <- factor(heatmap_df[["Locus"]], labels = loci_names)
heatmap_df[["Type"]] <- 'Neutral'
heatmap_df[["Type"]][which(heatmap_df[["Locus"]] %in% immune_marker_names)] <- 'Immune'

## sort by species
heatmap_df[["Species"]] <- factor(heatmap_df[["Species"]],
                          levels = c("Fur seal", "Grey seal",
                                     "Northern Elephant seal"),
                          labels = c("Antarctic fur seal", "Grey seal",
                                     "Northern Elephant seal"))

## define colors for marker types
col_key <- ifelse(levels(heatmap_df[["Locus"]]) %in% immune_marker_names,
                  "#C1403D", "#0294A5")
```

```r
plot <- ggplot(data = heatmap_df, aes(x = Species, y = Locus, fill = Alleles)) +
  theme_classic() +
  geom_tile(colour = "Black", size = .75) +
  scale_fill_viridis_c(name = "Alleles", na.value = "Grey75") +
  scale_x_discrete(expand = c(0,0)) +
  theme(
    plot.margin = margin(t = 5, r = 25, b = 5, l = 15, unit = "mm"),
    legend.position = c(1,1),
    legend.justification = c(0, 1),
    legend.direction = "vertical",
    legend.margin = margin(0,0,0,5, "mm"),
    axis.text.y = element_text(hjust = 0, colour = col_key, size = 8),
    axis.line.x = element_blank(),
    axis.text.x = element_text(size = 10)) +
  xlab("Species") +
  ylab("")

ggsave(plot,
       filename = 'HeatmapLoci.tiff',
       width = 6,
       height = 9,
       units = "in",
       dpi = 300)
```

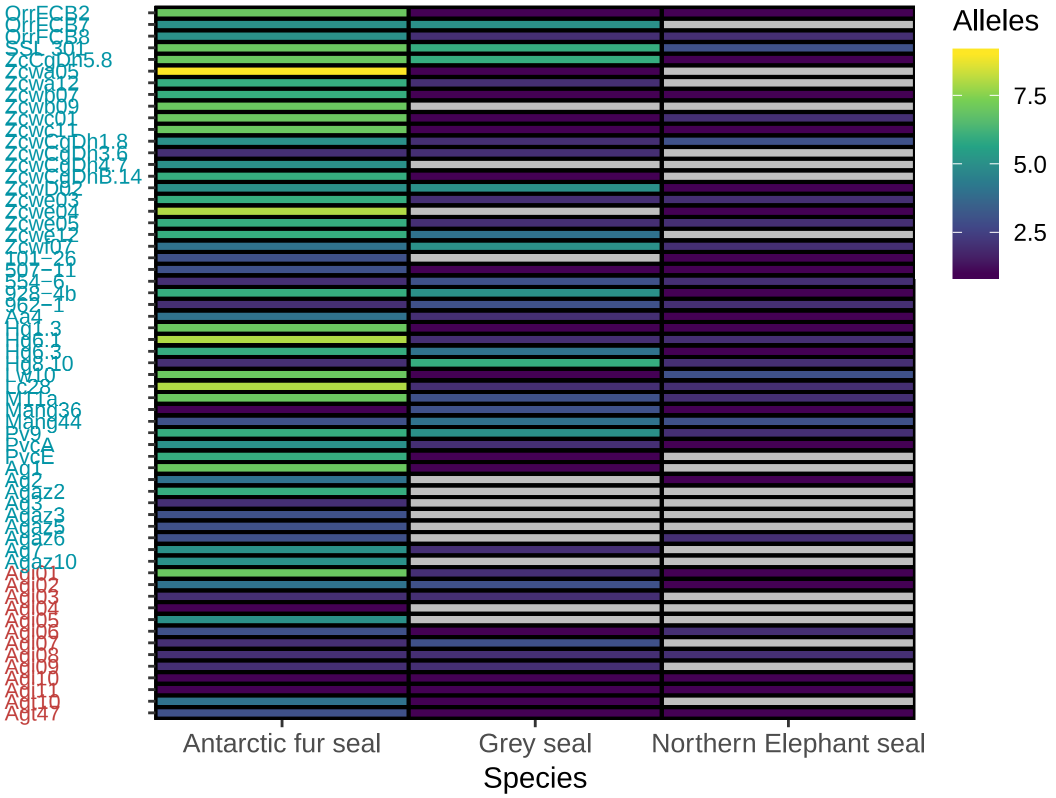

The heatmap above shows several patterns which are tested statistically next.

```
## get raw data again
genotypes_raw <- readxl::read_xlsx("data/data.xlsx", skip = 1)[, c(3, 8:ncol(seals))]

## extract genotypes
marker_geno <- apply(genotypes_raw[,-1], 2, as.character)

## collapse information for each locus in one column
marker_geno <- lapply(seq(1, ncol(marker_geno), 2), function(x) {
  marker_geno[,x:(x + 1)] %>%
    apply(., 1, paste0, collapse = "/")
}) %>%
  do.call("cbind",.) %>%
  ## rename loci
  set_colnames(x = ., value = paste0("Locus", 1:61))

## set missing data to NA
marker_geno[which(marker_geno == "NA/NA")] <- NA

## create GENIND for Antarctic fur seal alone
genind_afs <- adegenet::df2genind(marker_geno[1:78,], ploidy = 2, sep = "/")
```

```
## extract allele numbers for both marker types
immune_afs <- genind@loc.n.all[1:13]
mean(immune_afs)
sd(immune_afs)
```

```r
neutral_afs <- genind@loc.n.all[14:61]
mean(neutral_afs)
sd(neutral_afs)

## compare marker types
wilcox.test(immune_afs, neutral_afs, paired = F)
```

```r
## compare neutral markers by origin
neutral_afs <- genind@loc.n.all[14:22]
mean(neutral_afs)
sd(neutral_afs)

neutral_others <- genind@loc.n.all[23:61]
mean(neutral_others)
sd(neutral_others)

## compare by marker
wilcox.test(neutral_afs, neutral_others, paired = F)
```

```r
## cross-amplification
immune <- dplyr::filter(heatmap_df, Species != "Antarctic fur seal",
                        Type == "Immune")[["Alleles"]]
immune <- ifelse(is.na(immune), 0, 1) # check if amplified
mean(immune) ## cross-amplification rate

neutral <- dplyr::filter(heatmap_df, Species != "Antarctic fur seal")[27:44, "Alleles"]
neutral <- ifelse(is.na(neutral), 0, 1) # check if amplified
mean(neutral) ## cross-amplification rate


wilcox.test(neutral, immune, paired = F)
```

(b) FDR Correction

After receiving HWE values, apply a false discovery rate correction for a table of p-values.

```r
pval <-
  read.table("data/pvalues.txt",header = F, sep = ",") %>%
  as.vector() %>% .[[1]]
qobj <- qvalue(pval)
qvalues <- qobj$qvalues
pi0 <- qobj$pi0
lfdr <- qobj$lfdr

summary(qobj)
df <- data.frame(p = qobj$pvalues,
                 q = qobj$qvalues)
## view(df)
```

(c) Sensitivity of loci number on estimates of g_2

Here, we repeat the estimation of g_2 for each marker type and for the entire dataset

```r
g2_neutral_resampled <- pbapply::pblapply(seq(4, 48, 4), function(x) {
  subs <- lapply(1:100, function(y) {
    rand <- sample(1:48, x, replace = FALSE)
    loci <- neutral_markers[, rand]
    g2 <- g2_microsats(loci, nperm = 0, nboot = 9999, verbose = F)
    df <- data.frame(bs = g2$g2_boot,
                     lcl = g2$CI_boot[[1]],
                     ucl = g2$CI_boot[[2]],
                     g2  = g2$g2,
                     p = g2$p_val)
    return(df[1,])
  }) %>% do.call("rbind", .)
  return(data.frame(g2 = mean(subs$g2),
                    lcl = confidence_interval(subs$g2)[1],
                    ucl = confidence_interval(subs$g2)[2]))
}) %>%  do.call("rbind", .)
g2_neutral_resampled$loci <- seq(4, 48, 4)
```

```r
## load saved dataset
load("data/g2_neutral_resampled.RData")

g2_neutral_resampled_plot <-
  ggplot(data = g2_neutral_resampled, aes(x = loci, y = g2)) +
  geom_line() +
  geom_point(size = 1.5) +
  geom_errorbar(aes(ymin = lcl,
                    ymax = ucl),
                width = 0.8, alpha = 0.7, size = 0.8, colour = "black") +
  geom_hline(yintercept = 0, linetype = "dotted") +
  theme_classic() +
  theme(legend.position = "none",
        panel.border = element_blank(),
        strip.background = element_blank(),
        text = element_text(size = 12),
        aspect.ratio = 1,
        axis.title.y = element_text(face = "italic"),
        plot.margin = grid::unit(c(2,2,2,2), 'mm')) +
  xlab("Number of loci") +
  labs(y = expression(italic(g)["2"])) +
  scale_x_continuous(expand = c(0,0), limits = c(0, 50))
```

```r
g2_neutral_resampled_plot
```

Microsatellite development

Identifying microsatellites within the transcriptome

Short tandem repeats were identified within the Antarctic fur seal transcriptome assembly using the script `misa.pl`. The required initiation file called `misa.ini` is available in the folder `data` and defines the minimum number of five repeats for di-, tri- and tetranucleotide motifs. *As already mentioned before, MISA needs to be downloaded by the user*

```
# identify microsats
perl misa.pl arc_gaz_transcriptome.fasta
```

The code above generates one ouput file `arc_gaz_transcriptome.fasta.misa` containing a total of 2577 microsatellites found within the transcriptome. The resulting data table was subsequently reformatted for further filtering steps.

```
## read the data
data <- readLines("data/arc_gaz_transcriptome.fasta.misa")

## create a matrix
microsats_table <- matrix(ncol = length(strsplit(data[1], split = "\t")[[1]]),
                          nrow = length(data))
for (i in 1:length(data)) {
microsats_table[i,1:length(strsplit(data[i], split = "\t")[[1]])] <-
  strsplit(data[i], split = "\t")[[1]]
}
microsats_table <- as.data.frame(microsats_table[2:nrow(microsats_table),])

## add column names
names(microsats_table) <-
  c('contig.name', 'ssr.no','ssr.type','ssr.seq','ssr.size','ssr.start','ssr.end')

## read contig length information
contig_length <- read.table("data/transcriptlength.txt", header = T)
names(contig_length) <- c('MatchID', 'contig.length')

## set to character
microsats_table[["contig.name"]] <-
  as.character(microsats_table[["contig.name"]])
contig_length[["MatchID"]] <-
  as.character(contig_length[["MatchID"]])

## correct 'MatchID' for cross-referencing
microsats_table[["MatchID"]] <- NA
for (i in 1:nrow(microsats_table)) {
  # discard chunk following the underscore (e.g. 4708387_length... becomes 4708387)
  microsats_table$MatchID[i] <-
   strsplit(microsats_table$contig.name[i],split = "_")[[1]][1]
  microsats_table$MatchID[i] <-
    strsplit(microsats_table$MatchID[i],split = " ")[[1]][1]
}

## correct contig_length
for (i in 1:nrow(contig_length)) {
  # remove everything after the underscore, see above
  contig_length$MatchID[i] <-
    strsplit(contig_length$MatchID[i],split = "_")[[1]][1]
}

## merge data frames
microsats_table <-
dplyr::left_join(microsats_table,contig_length, by = "MatchID")
```

```
## some data class conversions
microsats_table[["ssr.start"]] <-
  as.numeric(as.character(microsats_table[["ssr.start"]]))
microsats_table[["ssr.end"]] <-
  as.numeric(as.character(microsats_table[["ssr.end"]]))
microsats_table[["contig.length"]] <-
  as.numeric(as.character(microsats_table[["contig.length"]]))

## export Supplementary Table S1
write.csv(microsats_table, file = "data/Supplementary Table S1.csv", row.names = F)

## summary of microsatellite types
summary(microsats_table$ssr.type)
```

```
##    c   c*   p2   p3   p4
##  211    4 1687  595   80
```

Filtering Microsatellites

Among the 2577 identified microsatellites, there are some compound microsatellites as well as repeats that do not offer adequate flanking sites for primer design. These were discarded next.

```
## remove compound microsats
microsats_table <-
  subset(microsats_table, microsats_table[["ssr.type"]] != 'c')
microsats_table <-
  subset(microsats_table, microsats_table[["ssr.type"]] != 'c*')

## selection based on flanking sites
microsats_table[["temp"]] <-
  rep(1,nrow(microsats_table)) # flag for removal
for (i in 1:nrow(microsats_table)) {
  # inspect flanking site upstream
if (microsats_table[["ssr.start"]][i] <= 100) {
      microsats_table[["temp"]][i] <- 0
      # inspect flanking site downstream
} else if ((microsats_table[["contig.length"]][i] -
            microsats_table[["ssr.end"]][i]) <= 100) {
    microsats_table[["temp"]][i] <- 0
  }
}

## Remove flagged microsats
microsats_table <- subset(microsats_table, microsats_table[["temp"]] != 0)
microsats_table <- microsats_table[,1:9]
```

After the above filtering 1580 microsatellites were retained. Now, we selected microsatellites that are associated to immunity based on Gene Ontology Gene annotations.

```
## Keywords including 'immune*'
annotation <- readLines("data/arc_gaz_transcriptome_annotations.txt")
annotations <- matrix(ncol = 18, nrow = length(annotation))

## fill table
```

```r
for (i in 1:nrow(annotations)) {
annotations[i, 1:length(strsplit(annotation[i], split = "\t")[[1]])] <-
  strsplit(annotation[i], split = "\t")[[1]]
}
annotations <- data.frame(annotations)[-1,]
annotations <- annotations[, c(1,14:18)]
names(annotations) <- c('MatchID','goTerm','cellular.components','biological.processes',
                        'molecular.functions','keywords')

annotations[["MatchID"]] <- as.character(annotations[["MatchID"]])
for (i in 1:nrow(annotations)) {
  annotations[["MatchID"]][i] <-
    strsplit(annotations[["MatchID"]][i],split = "_")[[1]][1]
}

annotations.extd <- dplyr::left_join(microsats_table, annotations,by = 'MatchID')

immuneTable2 <- data.frame(annotations.extd) %>% # Check for matches with keywords
  dplyr::filter(grepl('immun*', keywords))

ImmuneMarker_Keywords <- immuneTable2 # 13 within just keywords
```

For 13 microsatellites, we found a match to the term 'immun*' under the keywords of the GO annotations. To increase the number of suitable microsatellites, we repeated the initial search to all categories of the GO annotations with an extended list of search terms shown below.

```r
## define list of keywords
immune <- c('immun*', 'antigen', 'chemokine', 'T cell',
            'MHC', 'Antibody', 'histocompatibility',
            'Interleukin', 'Leucocyte', 'Lymphocyte')

immuneLines <- NULL
for (i in immune) {
  immuneLines <- c(immuneLines, annotation[grep(i, annotation, ignore.case = T)])
}

immuneTable <- matrix(ncol = 18, nrow = length(immuneLines))

for (i in 1:length(immuneLines)) {
immuneTable[i,1:length(strsplit(immuneLines[i], split = "\t")[[1]])] <-
  strsplit(immuneLines[i], split = "\t")[[1]]
}
immuneTable <- data.frame(immuneTable)[,c(1,10,14:18)]
names(immuneTable) <-
  c('MatchID','geneID','goTerm','cellular.components','biological.processes',
                        'molecular.functions','keywords')

immuneTable[["MatchID"]] <-
  as.character(immuneTable[["MatchID"]])
for (i in 1:nrow(immuneTable)) {
  immuneTable[["MatchID"]][i] <-
    strsplit(immuneTable[["MatchID"]][i],split = "_")[[1]][1]
}
```

```r
ImmuneMarker_whole_file <-
  unique(dplyr::inner_join(microsats_table, immuneTable, by = "MatchID"))

## write to file
write.csv2(ImmuneMarker_whole_file, file = "data/immune_microsats_raw.csv", row.names = F)
```

The extended search yielded a total of 137 microsatellites. The entire list is shown below.

Table 1: Annotated microsatellites

	Contig	Motif	Start	End	Gene ID
1	AgU000001_v1.1	(CA)5	1586	1595	VWF_CANLF
2	AgU000018_v1.1	(AC)5	4757	4766	AGRF5_HUMAN
4	AgU000018_v1.1	(TG)5	5364	5373	AGRF5_HUMAN
6	AgU000026_v1.1	(GT)5	3260	3269	BMR1A_HUMAN
8	AgU000026_v1.1	(CA)5	3970	3979	BMR1A_HUMAN
10	AgU000033_v1.1	(CT)5	1804	1813	RORA_MOUSE
12	AgU000033_v1.1	(AAT)5	3514	3528	RORA_MOUSE
14	AgU000038_v1.1	(TA)5	3523	3532	IL6RB_HUMAN
17	AgU000053_v1.1	(AG)6	3638	3649	AKAP9_HUMAN
18	AgU000073_v1.1	(TA)6	2171	2182	TGFR2_HUMAN
20	AgU000074_v1.1	(AC)8	4025	4040	CD302_PIG
21	AgU000087_v1.1	(ATT)6	3163	3180	ITAV_BOVIN
22	AgU000123_v1.1	(CT)5	511	520	NCKP1_HUMAN
23	AgU000160_v1.1	(TC)6	2967	2978	EMP2_BOVIN
24	AgU000254_v1.1	(TC)13	1338	1363	CD44_CANLF
25	AgU000356_v1.1	(GA)5	1841	1850	IL3RB_HUMAN
27	AgU000367_v1.1	(AC)5	2501	2510	PSA_HUMAN
29	AgU000376_v1.1	(CAG)5	458	472	EGR1_HUMAN
31	AgU000376_v1.1	(CCT)5	805	819	EGR1_HUMAN
33	AgU000386_v1.1	(TC)5	1928	1937	EZRI_HUMAN
36	AgU000395_v1.1	(CAG)5	593	607	TISD_HUMAN
37	AgU000395_v1.1	(CGC)5	1044	1058	TISD_HUMAN
38	AgU000395_v1.1	(AAC)5	2333	2347	TISD_HUMAN
39	AgU000416_v1.1	(AC)5	2494	2503	RAB5B_PONAB
40	AgU000523_v1.1	(GCG)7	2676	2696	MAPK2_HUMAN
42	AgU000542_v1.1	(AG)5	662	671	ERBB3_HUMAN
43	AgU000543_v1.1	(TG)7	1250	1263	G9L1E5_MUSPF
44	AgU000568_v1.1	(CT)7	1660	1673	IL33_CANLF
45	AgU000696_v1.1	(CG)5	2544	2553	SOCS3_HUMAN
46	AgU000706_v1.1	(GT)15	1718	1747	PTPRJ_HUMAN
48	AgU000706_v1.1	(TA)6	1865	1876	PTPRJ_HUMAN
50	AgU000892_v1.1	(CA)6	1013	1024	SDCB1_HUMAN
52	AgU000895_v1.1	(GAT)5	1865	1879	VAMP7_HUMAN
53	AgU000982_v1.1	(CT)6	1390	1401	ERRFI_HUMAN
54	AgU001017_v1.1	(TCC)5	1891	1905	MSH6_HUMAN
55	AgU001054_v1.1	(TC)5	964	973	SDF1_HUMAN
58	AgU001075_v1.1	(AC)6	1677	1688	SIN3A_HUMAN
59	AgU001075_v1.1	(TC)6	2012	2023	SIN3A_HUMAN
60	AgU001116_v1.1	(GT)5	1753	1762	MYLK_SHEEP

Table 1: Annotated microsatellites *(continued)*

	Contig	Motif	Start	End	Gene ID
61	AgU001116_v1.1	(GT)5	2153	2162	MYLK_SHEEP
62	AgU001227_v1.1	(GA)6	1585	1596	UBA3_HUMAN
63	AgU001338_v1.1	(GAT)7	1822	1842	VAMP3_HUMAN
64	AgU001432_v1.1	(AT)8	1652	1667	FOXC1_HUMAN
65	AgU001679_v1.1	(GA)5	1766	1775	TOPRS_HUMAN
66	AgU001875_v1.1	(TA)10	1659	1678	ACKR3_CANLF
67	AgU001893_v1.1	(AG)5	504	513	PDPK1_HUMAN
69	AgU002020_v1.1	(TC)5	1366	1375	TF65_MOUSE
71	AgU002096_v1.1	(CTG)5	676	690	M3YA16_MUSPF
73	AgU002096_v1.1	(GA)5	1309	1318	M3YA16_MUSPF
75	AgU002160_v1.1	(AAG)6	1065	1082	PK3CB_MOUSE
76	AgU002268_v1.1	(GCG)5	1137	1151	CEBPB_HUMAN
79	AgU002404_v1.1	(GC)5	1363	1372	NFKB2_HUMAN
80	AgU002472_v1.1	(TAAA)5	539	558	ANKR1_HUMAN
81	AgU002542_v1.1	(AC)8	301	316	PAR1_HUMAN
82	AgU002562_v1.1	(AT)5	939	948	AP1AR_HUMAN
83	AgU002579_v1.1	(TG)5	1135	1144	AP2A1_HUMAN
85	AgU002812_v1.1	(CGG)9	163	189	TM131_HUMAN
86	AgU002813_v1.1	(TC)5	1011	1020	RIPK1_HUMAN
89	AgU002947_v1.1	(CT)5	1509	1518	NCK1_HUMAN
91	AgU003069_v1.1	(CT)5	688	697	NR4A1_BOVIN
92	AgU003233_v1.1	(AG)5	429	438	KSYK_PIG
93	AgU003302_v1.1	(GT)5	114	123	TNF13_HUMAN
94	AgU003381_v1.1	(TTC)5	131	145	OTU7B_HUMAN
96	AgU003480_v1.1	(AC)5	217	226	M3K5_HUMAN
97	AgU003551_v1.1	(GCA)5	1297	1311	CD14_BOVIN
99	AgU003600_v1.1	(AG)5	828	837	CY24B_HUMAN
102	AgU003731_v1.1	(TATT)6	1223	1246	IL1B_EUMJU
104	AgU003752_v1.1	(GA)5	687	696	DICER_HUMAN
105	AgU003880_v1.1	(AC)12	504	527	SNAI2_MOUSE
107	AgU004117_v1.1	(TA)5	1207	1216	I23O1_HUMAN
110	AgU004295_v1.1	(TC)5	1124	1133	HOIL1_HUMAN
111	AgU004366_v1.1	(CTC)6	223	240	RAGE_BOVIN
112	AgU004826_v1.1	(AT)7	886	899	FBX9_HUMAN
114	AgU005175_v1.1	(AT)7	304	317	ID2_PONAB
115	AgU005564_v1.1	(AG)5	1082	1091	TRIM5_ATEGE
116	AgU005573_v1.1	(CCG)5	193	207	TNR1A_HUMAN
117	AgU005575_v1.1	(GA)5	115	124	MEF2C_PONAB
119	AgU005648_v1.1	(GCT)7	207	227	PVRL2_HUMAN
121	AgU005740_v1.1	(GC)5	248	257	CD34_CANLF
123	AgU006059_v1.1	(CAT)5	397	411	PSA1_HUMAN
127	AgU006102_v1.1	(GT)5	807	816	SEM3C_PONAB
128	AgU006175_v1.1	(GA)6	227	238	F6PLB9_CANLF
131	AgU006223_v1.1	(TA)5	507	516	MP2K3_HUMAN
132	AgU006292_v1.1	(AT)8	503	518	NPTN_MOUSE
133	AgU006300_v1.1	(CG)5	544	553	TRPM4_HUMAN

Table 1: Annotated microsatellites *(continued)*

	Contig	Motif	Start	End	Gene ID
135	AgU006317_v1.1	(TA)9	797	814	CXL10_CANLF
138	AgU006325_v1.1	(TA)5	102	111	NPC1_HUMAN
139	AgU006325_v1.1	(CA)5	539	548	NPC1_HUMAN
140	AgU006358_v1.1	(GA)5	215	224	PTMS_HUMAN
141	AgU006358_v1.1	(GGC)5	900	914	PTMS_HUMAN
142	AgU006421_v1.1	(AG)8	311	326	CLC2D_HUMAN
143	AgU007141_v1.1	(GCT)5	341	355	ROBO4_HUMAN
144	AgU007556_v1.1	(AG)6	107	118	BST2_HUMAN
146	AgU007808_v1.1	(TC)5	678	687	AKIP1_HUMAN
147	AgU007843_v1.1	(CA)5	332	341	SMAD3_RAT
150	AgU007845_v1.1	(CATT)6	463	486	TNR12_HUMAN
151	AgU008174_v1.1	(CCT)5	147	161	CD2B2_MOUSE
152	AgU008391_v1.1	(CA)20	338	377	TNR1A_HUMAN
153	AgU009399_v1.1	(CA)5	665	674	UFO_MOUSE
156	AgU009504_v1.1	(TGC)5	440	454	EP300_HUMAN
159	AgU009791_v1.1	(AT)5	127	136	Q7Z5E4_HUMAN
160	AgU010008_v1.1	(CA)8	318	333	STA5A_HUMAN
162	AgU010547_v1.1	(AT)6	455	466	WASL_MOUSE
163	AgU010559_v1.1	(AT)6	250	261	CCL20_BOVIN
167	AgU010620_v1.1	(TG)5	309	318	AACS_RAT
168	AgU011733_v1.1	(CA)5	418	427	UB2L6_HUMAN
169	AgU011784_v1.1	(GC)5	419	428	ZN580_MOUSE
170	AgU013299_v1.1	(AC)5	546	555	RN125_MACFA
171	AgU013484_v1.1	(TC)5	389	398	DYHC1_HUMAN
173	AgU013617_v1.1	(TC)5	295	304	MARH7_HUMAN
174	AgU013753_v1.1	(TTC)5	484	498	MYH10_MOUSE
175	AgU013922_v1.1	(GT)5	310	319	ICAM3_PANTR
176	AgU014161_v1.1	(CT)5	304	313	CSPG2_BOVIN
177	AgU014501_v1.1	(GA)5	300	309	NKAP_HUMAN
178	AgU014501_v1.1	(AGA)6	445	462	NKAP_HUMAN
179	AgU032052_v1.1	(AT)6	1875	1886	SKAP2_HUMAN
180	AgU032202_v1.1	(AG)5	799	808	LEG3_CANLF
181	AgU032055_v1.1	(TA)5	524	533	TXNIP_HUMAN
182	AgU032268_v1.1	(AC)21	553	594	CD59_PIG
183	AgU025816_v1.1	(GGA)6	126	143	HS90A_HUMAN
185	AgU032568_v1.1	(CCT)6	365	382	KIF3B_HUMAN
187	AgU032760_v1.1	(AGA)6	122	139	CHD7_HUMAN
188	4741325_length_871_cvg_4.5_tip_1	(CA)5	760	769	GCSAM_HUMAN
189	4744327_length_942_cvg_8.2_tip_1	(AG)5	120	129	IFIH1_HUMAN
190	4744731_length_953_cvg_17.8_tip_0	(GA)6	219	230	CD20_CANLF
192	4744731_length_953_cvg_17.8_tip_0	(TTC)5	466	480	CD20_CANLF
194	4746463_length_1006_cvg_3.8_tip_1	(GGC)6	102	119	EGR2_PIG
195	4750219_length_1140_cvg_4.7_tip_1	(CT)5	568	577	SNAI1_HUMAN
196	4750387_length_1146_cvg_5.5_tip_1	(AAG)5	530	544	DAB2P_HUMAN
198	4750419_length_1148_cvg_8.7_tip_1	(CA)5	901	910	GAB2_HUMAN
200	4750933_length_1171_cvg_8.8_tip_1	(TC)5	945	954	DYH7_HUMAN

Table 1: Annotated microsatellites *(continued)*

	Contig	Motif	Start	End	Gene ID
201	4751237__length__1187__cvg__6.3__tip__1	(GA)5	192	201	SRC__HUMAN
204	4751391__length__1195__cvg__12.9__tip__0	(TC)5	704	713	MEFV__MOUSE
206	4753675__length__1332__cvg__9.9__tip__1	(TC)6	418	429	E2AK3__HUMAN
207	4754597__length__1401__cvg__5.7__tip__1	(GA)5	477	486	MYOM1__MOUSE
208	4755571__length__1487__cvg__10.6__tip__1	(AG)5	1029	1038	AGRA3__HUMAN
209	4756187__length__1545__cvg__8.7__tip__1	(CA)7	319	332	TNR9__HUMAN

Designing primers

For all of the 137 we developed oligonucleotide primers using the primer design tool primer3. In order to use the command line interface, the list of microsatellites should be re-formatted accordingly.

```
## list of microsatellites
data <- read.csv(file = "data/immune_microsats_raw.csv", sep = ';')[,1:7]
names(data) <- c('ID','SSR nr.','SSR type','SSR','size','start','end')
data[["ID"]] <- as.character(data[["ID"]])

for (i in 1:nrow(data)) {
  if ((nchar(data[["ID"]][i]) > 20)) {
    data[["ID"]][i] <-
      paste0(strsplit(data[["ID"]][[i]],split = "_")[[1]][1]," ",
             strsplit(data[["ID"]][[i]],split = "_")[[1]][2]," ",
             strsplit(data[["ID"]][[i]],split = "_")[[1]][3]," ",
             strsplit(data[["ID"]][[i]],split = "_")[[1]][4],"_",
             strsplit(data[["ID"]][[i]],split = "_")[[1]][5],"_",
             strsplit(data[["ID"]][[i]],split = "_")[[1]][6],"_",
             strsplit(data[["ID"]][[i]],split = "_")[[1]][7])
  }
}
write.table(row.names = FALSE, quote = FALSE,x = data,
            sep = "\t",file = 'data/arc_gaz_transcriptome.fasta.misa2')
```

Invoke primer3 for primer design

```
perl p3_in_fur_seal.pl arc_gaz_transcriptome.fasta.misa2
primer3_core <arc_gaz_transcriptome.fasta.p3in> arc_gaz_transcriptome.fasta.p3out
```

Overview of initially tested microsatellites

The table below summarises the results of testing 96 primers on 12 Antarctic fur seal indiviudals. See the manuscript for further details.

Table 2: Overview microsatellite testing. Primers used in the present study are named Agi01-Agi11

Contig	Gene ID	Motif	Marker ID	Forward primer 5'-3'	Reverse primer 5'-3'	PCR result
AgU000073	TGFR2_HUMAN	(TA)6	NA	GAAGCATTCTAGGCCTTTGACA	GAGCTCTCCAAACAAACCAATT	Monomorph
AgU000395	TISD_HUMAN	(AAC)5	Agi03	GCCTTGATTGTAGTCCTCAGC	GAACTAAGCTCTGCCCAAGG	Polymorph
AgU000568	IL33_CANLF	(CT)7	NA	GAGCCTGCTTCTCCCTCTG	TCCCTGAAGCATAGTGTCAGA	Failed
AgU000706	PTPRJ_HUMAN	(GT)15	Agi01	GGTTGGCATTTTATGTGTGTCC	TGCAGAGAGACTAAAGCCAGT	Polymorph
AgU000982	ERRFI_HUMAN	(CT)6	NA	CAGACTTTTCTCCAACGCCA	TGAAGCGCAAACATCTGTCC	Monomorph
AgU001227	UBA3_HUMAN	(GA)6	NA	TGGGGTTGGTACTTGTAAGCA	TGGGTGCTCACATGAAAACTG	Monomorph
AgU001338	VAMP3_HUMAN	(GAT)7	NA	CTGGGGCTACACTGGTTCTT	GGAGTTAGACGATCGTGCAG	Monomorph
AgU001875	ACKR3_CANLF	(TA)10	NA	GGCTAGTTGGATTTCAGTTTTGA	CTGTTCCATATCCCATGCCG	Monomorph
AgU003551	CD14_BOVIN	(GCA)5	NA	CAGAAGCAGCGGAAATCCTC	ACGTGTGTGGAGCCTAGAAA	Monomorph
AgU004366	RAGE_BOVIN	(CTC)6	Agi09	GGGGCTGATAGATGGGGTC	GAACTGTAGCCCTGGTCCTG	Polymorph
AgU006292	NPTN_MOUSE	(AT)8	NA	CTGCTGCCGTCTAGTGATGA	ACCAGAACTGCACGATTTCC	Monomorph
AgU006421	CLC2D_HUMAN	(AG)8	NA	GCCAACTATATACAAAGGGCGT	GCTTAACCAACTGAGCCACC	Failed
AgU007843	SMAD3_RAT	(CA)5	NA	ACGGAGAAGTGGGAATAACAGA	CACTGATGTCTTGTTGGGCA	Monomorph
AgU007845	TNR12_HUMAN	(CATT)6	NA	ATCCAGTGACAGTGAGAGCC	GCCTTGGAGAGCTGATTCAC	Monomorph
AgU008391	TNR1A_HUMAN	(CA)20	NA	CCCTATCTCTGCAGCCACAA	ATGCCCTTCGGACCCTTTT	Monomorph
AgU013617	MARH7_HUMAN	(TC)5	NA	TGGTCTTGCTCCCTGTGAAT	GTTCCCAGATCTTCATCAATGGT	Failed
AgU014501	NKAP_HUMAN	(GA)5	NA	TCTGACGAACACACACCAGT	TCATCGCTGGAGTCTGAGTC	Monomorph
AgU032055	TXNIP_HUMAN	(TA)5	NA	TGATAGCAGCAACCCTTCTCA	TCATGTGACTCCTTGGAATGG	Monomorph
AgU032268	CD59_PIG	(AC)21	NA	CTGCCAGACACCAGCTAGTT	ATCCTCTCCCTTTATGGCCC	Failed
AgU025816	HS90A_HUMAN	(GGA)6	NA	GAAGAGAAGGAGCCCGATGA	TGCCAAGTGATCTTCCCAGT	Failed
AgU003233	KSYK_PIG	(AG)5	NA	GTCATGTCCCGCACGAGG	GCTGCGCAACTACTACTACG	Monomorph
AgU003480	M3K5_HUMAN	(AC)5	NA	CTGCTTCTCGGATTCTGCAC	CTGTTGCACTTCGGCCAAAT	Monomorph
AgU005564	TRIM5_ATEGE	(AG)5	NA	GAAAGAGAGCAGCATGACGG	AAGACACTCAGGGGCACATG	Monomorph
AgU006175	F6PLB9_CANLF	(GA)6	Agi02	GGACTCCTTCAAGTTCGAATTTG	GAACACATCAGCTTGCCCTG	Polymorph
AgU006300	TRPM4_HUMAN	(CG)5	NA	AACGCTGTGTCCACCTTTTG	GCTCCGCCCCTTATCATCAT	Monomorph
AgU007556	BST2_HUMAN	(AG)6	NA	ACAGATGTTCTTCCCCTTAGAGA	GTGCCTCCATTGGTTAAGCG	Monomorph
AgU009399	UFO_MOUSE	(CA)5	NA	CCACTTGACTGGCATCTTGG	ATGCTGGTGAAGTTCATGGC	Failed
AgU013299	RN125_MACFA	(AC)5	NA	AACGGCAAAGTGGACAGAAC	GCGAAATGAGGGCACACATA	Failed
AgU032202	LEG3_CANLF	(AG)5	Agi04	TGCTTTCCACTTTAACCCGC	CAGGTCATGATCCCAGGGTC	Polymorph
4744327	IFIH1_HUMAN	(AG)5	NA	CTTTTAGCCACAGGTCAGCC	ACTTCCCATGGTGCCTGAAT	Failed
4750387	DAB2P_HUMAN	(AAG)5	NA	GGGAGCACTTTGAGTTCCAC	ATGGTGATGGTCTGGTAGCG	Monomorph

Table 2: Overview microsatellite testing. Primers used in the present study are named Agi01-Agi11 *(continued)*

Contig	Gene ID	Motif	Marker ID	Forward primer 5'-3'	Reverse primer 5'-3'	PCR result
4751391	MEFV_MOUSE	(TC)5	NA	GGCTGCTGAGTCTGGATGAT	AGTCCTAGTGTCACGCTACG	Monomorph
AgU000074	CD302_PIG	(AC)8	NA	GCCATGTTAAAAGGTCCAGCA	GTGGATGATCTGTGAACAAGTGT	Monomorph
AgU000254	CD44_CANLF	(TC)13	Agi05	TCCTCTTCTTCCTCCTCTTCC	AGAAGTCCCATTGGTCCTGG	Polymorph
AgU000523	MAPK2_HUMAN	(GCG)7	NA	AGGCTCGACTTGACATGGAA	CATGCTGTCCAACTCCCAAG	Monomorph
AgU000543	G9L1E5_MUSPF	(TG)7	NA	GTCCCCAGCACAACTCTTCT	TCAGGAAAGAACGCCAAAGC	Monomorph
AgU001432	FOXC1_HUMAN	(AT)8	Agi07	TACATACATCCCCGTGAGCC	ATCCCTTTCCAACCCACAGT	Polymorph
AgU002542	PAR1_HUMAN	(AC)8	NA	TTCTACACCGCACTGCAAAC	ACGACAAGTCTGATTTGCATGT	Monomorph
AgU002812	TM131_HUMAN	(CGG)9	NA	AGGGTGGTCGAAGTCTTTGT	CAAGCAGAGCCAGCACAG	Failed
AgU003880	SNAI2_MOUSE	(AC)12	Agi06	TCTTCACTCCGGCTCCAAAT	TCCTCTCAATCTAGCTGTCAGT	Polymorph
AgU004826	FBX9_HUMAN	(AT)7	NA	GGCTTCACATCCAGTCCTCT	CCCTCCCCTGAAGCAAGTAA	Monomorph
AgU005175	ID2_PONAB	(AT)7	Agi08	CAGAAATACACATCTCTGCCACT	TTTCAAAGGTGGAGCGTGAA	Polymorph
AgU005648	PVRL2_HUMAN	(GCT)7	NA	GAGTAGAGCGGGCGGGAA	CACTCGGACTTGCACATCCT	Monomorph
AgU010008	STA5A_HUMAN	(CA)8	NA	GATCTGGAGAGCAAGCTGGT	AGGCTCGCTCTCATGAATGT	Monomorph
4756187	TNR9_HUMAN	(CA)7	NA	TCCGAACCAATGGAAAGTTTGT	CTTGTGGGAAAGGGGCATTT	Failed
4744731	CD20_CANLF	(GA)6	NA	TGACATGTTTTGCCTGCAGT	GTGTTCATAGCTTCCAAGAGACA	Failed
4746463	EGR2_PIG	(GGC)6	NA	GGCAGGTGGTGTGGGTTATA	CTCCACTCACTCCACTCTCC	Monomorph
4753675	E2AK3_HUMAN	(TC)6	NA	TGAGCCCTTTACTGTGCAGA	TTTCTCCTCCAAGACCGACC	Failed
AgU001075	SIN3A_HUMAN	(TC)6	NA	TCCTTCCTTTCTGTCTTTCTTGT	CTGTTTGTGCCGAGGGTAAG	Monomorph
AgU007141	ROBO4_HUMAN	(GCT)5	NA	TCCACGCCTAGCCTGCTG	CAGAAGTGATTGCTGGTGGG	Failed
AgU009791	Q7Z5E4_HUMAN	(AT)5	NA	CACAGGTAGAGAGCAAACAAGG	TTGCAGCTGGTTTTCGAGTT	Monomorph
AgU010559	CCL20_BOVIN	(AT)6	NA	AGCAAACACAGACACACACA	ATGGAATTGGACAGAGCCCA	Failed
AgU010620	AACS_RAT	(TG)5	NA	TTCCCCATGTTCTTCCCGG	AAGGCAAGATCGCTCCTCAG	Monomorph
AgU014161	CSPG2_BOVIN	(CT)5	NA	CGAATGCTTTAGATGGTCTGGG	GTGCCAGCTACCTCCTTTCT	Monomorph
AgU032568	KIF3B_HUMAN	(CCT)6	NA	CCTTCCTCCTCACCCTCTTC	AAGCCAAGGGTCAATGAGGA	Monomorph
AgU000053	AKAP9_HUMAN	(AG)6	NA	TTTTACACAGACGTTTTGCAATG	CTGCTGTCCCTGAATCTTACT	Monomorph
AgU000087	ITAV_BOVIN	(ATT)6	NA	TGAGAAACATTTGTGCGAGGG	TCAAAAGTCTTTCACAGCCCTC	Monomorph
AgU000160	EMP2_BOVIN	(TC)6	NA	TCCGAATGCCAGCCTTCATA	CGGCCTCATGTACCTGATCT	Monomorph
AgU000892	SDCB1_HUMAN	(CA)6	NA	CGTGTTTTATAGGCGCGCA	CTGTGTTAGAACCAGTCACCT	Monomorph
AgU002160	PK3CB_MOUSE	(AAG)6	NA	AGGTGTGGATAAGTTGGCTGA	TGAACAATCCCCGATGACCA	Monomorph
AgU003731	IL1B_EUMJU	(TATT)6	NA	TCTACTTACTCGGAGCCAGC	GATGCTTCTTGGCCCTCTTG	Monomorph
AgU010547	WASL_MOUSE	(AT)6	NA	TGCACACAATAACAGGGAGT	GGATGATGATGAATGGGAAGACT	Failed

Table 2: Overview microsatellite testing. Primers used in the present study are named Agi01-Agi11 *(continued)*

Contig	Gene ID	Motif	Marker ID	Forward primer 5'-3'	Reverse primer 5'-3'	PCR result
AgU032052	SKAP2_HUMAN	(AT)6	NA	TGCTGACGAGGTATCTGTGG	TCAGTACGTTCACAGCTAGAATC	Monomorph
AgU032760	CHD7_HUMAN	(AGA)6	NA	GCCCAGCTAGTGAAGAGTGA	GGTTCTTTCGGTTCCTTCGG	Monomorph
AgU000033	RORA_MOUSE	(AAT)5	NA	AGCTTACCAGGAAGCAAAGT	TGCTAGCGTGTTCACTGTTG	Monomorph
AgU000376	EGR1_HUMAN	(CAG)5	NA	TGGAAGAGATGATGCTGCTGA	TCAGGAAAAGACTCTGCGGT	Monomorph
AgU000895	VAMP7_HUMAN	(GAT)5	NA	TTCACACACTTTGGCCATGT	TCAGCGAGGAGAAAGATTGGA	Failed
AgU001017	MSH6_HUMAN	(TCC)5	Agi11	TGTCTCATGAGCGTGGACTT	GCCCTATGTGTCGTCCAGTA	Polymorph
AgU002096	M3YA16_MUSPF	(CTG)5	NA	GTGGATGAAGACCGGACTGA	AGACAACCTGACTGCCTTCA	Monomorph
AgU002268	CEBPB_HUMAN	(GCG)5	NA	TCCTCCTTCCGCTTGCAG	ACCTCTTCTCCGACGACTAC	Failed
AgU002472	ANKR1_HUMAN	(TAAA)5	NA	TGAATACCAGTGGCATCGAAG	CCAGCTCCTATCCACCTGTT	Monomorph
AgU003302	TNF13_HUMAN	(GT)5	NA	CCCTTCCAGCTCTTCAGTGA	GCAAGCGGAAAGAGAAGTCA	Monomorph
AgU005573	TNR1A_HUMAN	(CCG)5	NA	GATCTTCACCCCGGTCTCC	ACCAGTGCCGTAACCCTTAA	Failed
AgU006059	PSA1_HUMAN	(CAT)5	NA	TGTGCCTTTCTCTGTGGTCT	TTAAACATGGTCTGCGTGCC	Monomorph
AgU006358	PTMS_HUMAN	(GA)5	NA	GCCTTCTCCTCCACCTTCTC	TCTTCCAGAGACCCAGCTTG	Failed
AgU008174	CD2B2_MOUSE	(CCT)5	NA	CCCAGAGAGCCGATCCAAG	GGGTGAAGATTAGGGAGCGA	Monomorph
AgU009504	EP300_HUMAN	(TGC)5	NA	GGAACTGGTTATGGTTGGCC	TGCCGAACATGAACCCCA	Monomorph
AgU013753	MYH10_MOUSE	(TTC)5	NA	TCCAAGTCCTGAATATGCGC	CGAGCTGGAAGAGATGGAGA	Failed
AgU000001	VWF_CANLF	(CA)5	NA	GGGATTGGTCAGGGTCATCT	GGGCGGAAGGTCAATTGTAC	Monomorph
AgU002562	AP1AR_HUMAN	(AT)5	NA	AGTGGCTGCATGTAAAAGGA	GCACAATTGAGTAGATGACCCT	Monomorph
AgU000123	NCKP1_HUMAN	(CT)5	NA	GCTTCATTTTGTGCCATGGG	GTGACACAGCTGCCTCTTTG	Monomorph
AgU000356	IL3RB_HUMAN	(GA)5	NA	AATGTGCGTGTGTCTGTGTC	ACATGAGTGGGAGGAGGTCT	Monomorph
AgU000367	PSA_HUMAN	(AC)5	NA	AAAGGCAGGGTTTTAGCAGC	TCGGAAACCATACCCTGATGA	Failed
AgU000376	EGR1_HUMAN	(CCT)5	NA	TTTCTGCTCGTAGTCCTGCA	AGCTCTGCATGGGGAATCAT	Failed
AgU000416	RAB5B_PONAB	(AC)5	NA	GACTCTGAAGGACCCAGCTT	TGGGGAAAGATGCACAGAGA	Monomorph
AgU000542	ERBB3_HUMAN	(AG)5	NA	ACTAGCCAACGAGTTCACCA	CATCCTCCTCTGCCTCCAAG	Monomorph
AgU001054	SDF1_HUMAN	(TC)5	NA	CCCCTCATCCTCAGCTCTTC	CGCAGGATTGGACAACAGAC	Monomorph
AgU001116	MYLK_SHEEP	(GT)5	NA	ATGTGCATCAGTCAGGCCTT	CCTGCACTTTACAAACAGTGGA	Monomorph
AgU001679	TOPRS_HUMAN	(GA)5	NA	ACGAGATCTTGATCTGCTGGT	CAGATTCCCGTTCCCAGAGT	Monomorph
AgU001893	PDPK1_HUMAN	(AG)5	NA	TCAAAGAGAACAAGGTCCCGT	ACTCCAGAGCTGACACCATC	Failed
AgU002020	TF65_MOUSE	(TC)5	Agi10	CTTTGGGTAATGTCTTCTGGGG	GAAGCTGGAGGGTAGGGATG	Polymorph
AgU002404	NFKB2_HUMAN	(GC)5	NA	GCAGGTGATTGGTGAGGTTG	GTACAATGCGCGCCTGTT	Failed
AgU002579	AP2A1_HUMAN	(TG)5	NA	CCATCCAGGGGCTGTGTATT	CTGCTACCTGGTGTCCGG	Monomorph

Table 2: Overview microsatellite testing. Primers used in the present study are named Agi01-Agi11 *(continued)*

Contig	Gene ID	Motif	Marker ID	Forward primer 5'-3'	Reverse primer 5'-3'	PCR result
AgU002813	RIPK1_HUMAN	(TC)5	NA	TGAATGTCATTGCGGAAGGT	CTGATACACGTTCTCTGTCTGC	Monomorph
AgU002947	NCK1_HUMAN	(CT)5	NA	TGTCCATTGTAGCTACCCCG	AGTGTGCCAGATTCTGCATC	Failed
AgU003069	NR4A1_BOVIN	(CT)5	NA	TCAAGGTGTGGAGAAGTGGG	TTCTCACCCAGCCAGACGTA	Monomorph

***

```
## - Session info ----------------------------------------------------------
##  setting  value
##  version  R version 3.5.3 (2019-03-11)
##  os       Windows 10 x64
##  system   x86_64, mingw32
##  ui       RTerm
##  language (EN)
##  collate  English_United Kingdom.1252
##  ctype    English_United Kingdom.1252
##  tz       Europe/Berlin
##  date     2019-05-13
##
## - Packages --------------------------------------------------------------
##  ! package     * version  date       lib source
##    ade4        * 1.7-13   2018-08-31 [1] CRAN (R 3.5.1)
##    adegenet    * 2.1.1    2018-02-02 [1] CRAN (R 3.5.1)
##    AICcmodavg  * 2.2-1    2019-03-08 [1] CRAN (R 3.5.3)
##    ape           5.3      2019-03-17 [1] CRAN (R 3.5.3)
##    assertthat    0.2.0    2017-04-11 [1] CRAN (R 3.5.1)
##    boot          1.3-20   2017-08-06 [2] CRAN (R 3.5.3)
##    cellranger    1.1.0    2016-07-27 [1] CRAN (R 3.5.1)
##    class         7.3-15   2019-01-01 [2] CRAN (R 3.5.3)
##    classInt      0.3-1    2018-12-18 [1] CRAN (R 3.5.3)
##    cli           1.0.1    2018-09-25 [1] CRAN (R 3.5.1)
##    cluster       2.0.7-1  2018-04-13 [2] CRAN (R 3.5.3)
##    coda          0.19-2   2018-10-08 [1] CRAN (R 3.5.1)
##    codetools     0.2-16   2018-12-24 [2] CRAN (R 3.5.3)
##    colorspace    1.4-0    2019-01-13 [1] CRAN (R 3.5.3)
##    crayon        1.3.4    2017-09-16 [1] CRAN (R 3.5.1)
##    data.table    1.12.0   2019-01-13 [1] CRAN (R 3.5.3)
##    DBI           1.0.0    2018-05-02 [1] CRAN (R 3.5.1)
##    deldir        0.1-16   2019-01-04 [1] CRAN (R 3.5.2)
##    digest        0.6.18   2018-10-10 [1] CRAN (R 3.5.1)
##    dplyr         0.8.0.1  2019-02-15 [1] CRAN (R 3.5.3)
##    e1071         1.7-0.1  2019-01-21 [1] CRAN (R 3.5.3)
##    evaluate      0.13     2019-02-12 [1] CRAN (R 3.5.3)
##    expm          0.999-3  2018-09-22 [1] CRAN (R 3.5.1)
##    fansi         0.4.0    2018-10-05 [1] CRAN (R 3.5.1)
##    gdata         2.18.0   2017-06-06 [1] CRAN (R 3.5.0)
##    ggplot2     * 3.1.0    2018-10-25 [1] CRAN (R 3.5.1)
##    glue          1.3.1    2019-03-12 [1] CRAN (R 3.5.3)
##    gmodels       2.18.1   2018-06-25 [1] CRAN (R 3.5.1)
##    gridExtra   * 2.3      2017-09-09 [1] CRAN (R 3.5.1)
##    gtable        0.2.0    2016-02-26 [1] CRAN (R 3.5.1)
##    gtools        3.8.1    2018-06-26 [1] CRAN (R 3.5.0)
##    hms           0.4.2    2018-03-10 [1] CRAN (R 3.5.1)
##    htmltools     0.3.6    2017-04-28 [1] CRAN (R 3.5.1)
##    httpuv        1.5.0    2019-03-15 [1] CRAN (R 3.5.3)
##    httr          1.4.0    2018-12-11 [1] CRAN (R 3.5.3)
##    igraph        1.2.4    2019-02-13 [1] CRAN (R 3.5.3)
##    inbreedR    * 0.3.2    2016-09-09 [1] CRAN (R 3.5.1)
##    kableExtra  * 0.9.0    2018-05-21 [1] CRAN (R 3.5.1)
```

```
##    knitr         1.22     2019-03-08 [1] CRAN (R 3.5.3)
##    labeling      0.3      2014-08-23 [1] CRAN (R 3.5.0)
##    later         0.8.0    2019-02-11 [1] CRAN (R 3.5.3)
##    lattice       0.20-38  2018-11-04 [2] CRAN (R 3.5.3)
##    lazyeval      0.2.2    2019-03-15 [1] CRAN (R 3.5.3)
##    LearnBayes    2.15.1   2018-03-18 [1] CRAN (R 3.5.0)
##    lme4        * 1.1-21   2019-03-05 [1] CRAN (R 3.5.3)
##    magrittr    * 1.5      2014-11-22 [1] CRAN (R 3.5.1)
##    MASS          7.3-51.1 2018-11-01 [2] CRAN (R 3.5.3)
##    Matrix      * 1.2-15   2018-11-01 [2] CRAN (R 3.5.3)
##    mgcv          1.8-27   2019-02-06 [2] CRAN (R 3.5.3)
##    mime          0.6      2018-10-05 [1] CRAN (R 3.5.1)
##    minqa         1.2.4    2014-10-09 [1] CRAN (R 3.5.1)
##    munsell       0.5.0    2018-06-12 [1] CRAN (R 3.5.1)
##    nlme          3.1-137  2018-04-07 [2] CRAN (R 3.5.3)
##    nloptr        1.2.1    2018-10-03 [1] CRAN (R 3.5.1)
##    permute       0.9-5    2019-03-12 [1] CRAN (R 3.5.3)
##    pillar        1.3.1    2018-12-15 [1] CRAN (R 3.5.3)
##    pkgconfig     2.0.2    2018-08-16 [1] CRAN (R 3.5.1)
##    plyr          1.8.4    2016-06-08 [1] CRAN (R 3.5.1)
##    promises      1.0.1    2018-04-13 [1] CRAN (R 3.5.1)
##    purrr         0.3.2    2019-03-15 [1] CRAN (R 3.5.3)
##    qvalue      * 2.12.0   2018-05-01 [1] Bioconductor
##    R6            2.4.0    2019-02-14 [1] CRAN (R 3.5.3)
##    raster        2.8-19   2019-01-30 [1] CRAN (R 3.5.3)
##    Rcpp          1.0.1    2019-03-17 [1] CRAN (R 3.5.3)
##    readr         1.3.1    2018-12-21 [1] CRAN (R 3.5.3)
##    readxl      * 1.3.1    2019-03-13 [1] CRAN (R 3.5.3)
##    reshape2    * 1.4.3    2017-12-11 [1] CRAN (R 3.5.1)
##    rlang         0.3.1    2019-01-08 [1] CRAN (R 3.5.3)
##    rmarkdown     1.12     2019-03-14 [1] CRAN (R 3.5.3)
##    rstudioapi    0.9.0    2019-01-09 [1] CRAN (R 3.5.3)
##    rvest         0.3.2    2016-06-17 [1] CRAN (R 3.5.1)
##    scales        1.0.0    2018-08-09 [1] CRAN (R 3.5.1)
##    seqinr        3.4-5    2017-08-01 [1] CRAN (R 3.5.1)
##    sessioninfo   1.1.1    2018-11-05 [1] CRAN (R 3.5.1)
##    sf            0.7-3    2019-02-21 [1] CRAN (R 3.5.3)
##    shiny         1.2.0    2018-11-02 [1] CRAN (R 3.5.1)
##    sp            1.3-1    2018-06-05 [1] CRAN (R 3.5.1)
##    spData        0.3.0    2019-01-07 [1] CRAN (R 3.5.3)
##    spdep         1.0-2    2019-02-13 [1] CRAN (R 3.5.3)
##    stringi       1.4.3    2019-03-12 [1] CRAN (R 3.5.3)
##    stringr       1.4.0    2019-02-10 [1] CRAN (R 3.5.3)
##  R survival      2.43-3   <NA>       [2] <NA>
##    tibble        2.1.1    2019-03-16 [1] CRAN (R 3.5.3)
##    tidyselect    0.2.5    2018-10-11 [1] CRAN (R 3.5.1)
##    units         0.6-2    2018-12-05 [1] CRAN (R 3.5.1)
##    unmarked      0.12-3   2019-02-05 [1] CRAN (R 3.5.3)
##    utf8          1.1.4    2018-05-24 [1] CRAN (R 3.5.1)
##    vegan         2.5-4    2019-02-04 [1] CRAN (R 3.5.3)
##    VGAM          1.1-1    2019-02-18 [1] CRAN (R 3.5.3)
##    viridisLite   0.3.0    2018-02-01 [1] CRAN (R 3.5.1)
##    withr         2.1.2    2018-03-15 [1] CRAN (R 3.5.1)
##    xfun          0.5      2019-02-20 [1] CRAN (R 3.5.3)
```

```
##    xml2          1.2.0    2018-01-24 [1] CRAN (R 3.5.1)
##    xtable        1.8-3    2018-08-29 [1] CRAN (R 3.5.1)
##    yaml          2.2.0    2018-07-25 [1] CRAN (R 3.5.1)
##
## [1] C:/Users/MOMO/Documents/R/win-library/3.5
## [2] C:/Program Files/R/R-3.5.3/library
##
##  R -- Package was removed from disk.
```
