# Supplementary material for: Heterozygosity at neutral and immune loci is not associated with neonatal mortality due to microbial infection in Antarctic fur seals
Source: Ecol Evol. 2019 Jun 20;9(14):7985–96. doi: 10.1002/ece3.5317 (PMC6662382; doi:10.1002/ece3.5317)
Supplement: Supplementary file 4 [file ECE3-9-7985-s004.docx]

Supplementary Table S3. Summary of the 48 neutral loci and the two previously published immune loci (Hoffman and Nichols, 2011) genotyped in this study, including their polymorphism characteristics in 234 Antarctic fur seal individuals. “Mix” denotes the PCR mastermix into which each locus was multiplexed and “T_a_” denotes the annealing temperature used in the PCR. Hardy–Weinberg *p*-values are shown prior to table-wide false discovery rate (FDR) correction, with significant values highlighted in bold. None of these deviations remained significant after adjustment for the FDR.

| **Locus** | **Reference** | **Fluorescent label used** | **Mix** | T_a_ **(°C)** | **Number of alleles** | **H_0_** | **H_e_** | **HWE *p*-value** | **FDR *q*-value** |
| --- | --- | --- | --- | --- | --- | --- | --- | --- | --- |
| **Pv9** | (Allen *et al.*, 1995) | FAM | 1 | 53 | 10 | .739 | .742 | 0.234 | 0.646 |
| **Hg6.3** | (Allen *et al.*, 1995) | FAM | 1 | 53 | 13 | .850 | .863 | 0.559 | 0.744 |
| **Hg8.10** | (Allen *et al.*, 1995) | VIC | 1 | 53 | 4 | .423 | .422 | 0.245 | 0.646 |
| **Hg1.3** | (Gemmell *et al.*, 1997) | VIC | 1 | 53 | 13 | .842 | .864 | 0.055 | 0.577 |
| **M11a** | (Hoelzel *et al.*, 1999) | NED | 1 | 53 | 18 | .919 | .920 | 0.087 | 0.577 |
| **PvcA** | (Coltman, Bowen and Wright, 1996) | PET | 1 | 53 | 9 | .795 | .808 | 0.064 | 0.577 |
| **Zcwb07** | (Hoffman, Steinfartz and Wolf, 2007) | PET | 1 | 53 | 13 | .919 | .870 | 0.641 | 0.824 |
| **Agaz2** | (Hoffman, 2009) | PET | 1 | 53 | 9 | .842 | .799 | 0.253 | 0.646 |
| **Ag3** | (Hoffman, Dasmahapatra and Nichols, 2008) | FAM | 2 | 60 | 2 | .252 | .270 | 0.329 | 0.649 |
| **Agaz6** | (Hoffman, 2009) | FAM | 2 | 60 | 4 | .615 | .616 | 0.885 | 0.854 |
| **OrrFCB7** | (Buchanan *et al.*, 1998) | FAM | 2 | 60 | 11 | .872 | .848 | 0.856 | 0.854 |
| **Ag2** | (Hoffman, Dasmahapatra and Nichols, 2008) | VIC | 2 | 60 | 7 | .778 | .772 | 0.441 | 0.683 |
| **OrrFCB2** | (Buchanan *et al.*, 1998) | NED | 2 | 60 | 13 | .880 | .860 | 0.955 | 0.880 |
| **Lw10** | (Davis *et al.*, 2002) | NED | 2 | 60 | 17 | .919 | .908 | 0.040* | 0.577 |
| **ZcwCO1** | (Hoffman, Steinfartz and Wolf, 2007) | PET | 2 | 60 | 11 | .821 | .857 | 0.225 | 0.646 |
| **Agaz5** | (Hoffman, 2009) | PET | 2 | 60 | 3 | .509 | .532 | 0.176 | 0.646 |
| **ZcwDh.B14** | (Hernandez-Velazquez *et al.*, 2005) | PET | 2 | 60 | 6 | .778 | .766 | 0.477 | 0.694 |
| **Ssl301** | (Huebinger *et al.*, 2007) | FAM | 3 | 60 | 14 | .927 | .889 | 0.317 | 0.649 |
| **Ag7** | (Hoffman, Dasmahapatra and Nichols, 2008) | VIC | 3 | 60 | 7 | .752 | .756 | 0.817 | 0.854 |
| **ZcwDh4.7** | (Hernandez-Velazquez *et al.*, 2005) | VIC | 3 | 60 | 14 | .838 | .878 | 0.910 | 0.854 |
| **ZcwE05** | (Stoffel *et al.*, 2018) | NED | 3 | 60 | 9 | .748 | .791 | 0.130 | 0.591 |
| **Ag1** | (Hoffman, Dasmahapatra and Nichols, 2008) | PET | 3 | 60 | 10 | .889 | .873 | 0.907 | 0.854 |
| **OrrFCB8** | (Buchanan *et al.*, 1998) | PET | 3 | 60 | 8 | .773 | .787 | 0.912 | 0.854 |
| **ZcwF07** | (Hoffman, Steinfartz and Wolf, 2007) | FAM | 4 | 60 | 9 | .791 | .763 | 0.565 | 0.744 |
| **ZcwD02** | (Wolf *et al.*, 2006) | FAM | 4 | 60 | 15 | .833 | .852 | 0.304 | 0.646 |
| **ZcwCgDh1.8** | (Hernandez-Velazquez *et al.*, 2005) | VIC | 4 | 60 | 7 | .782 | .765 | 0.882 | 0.854 |
| **Aa4** | (Gemmell *et al.*, 1997) | VIC | 4 | 60 | 6 | .782 | .744 | 0.434 | 0.683 |
| **ZcwCgDh5.8** | (Hernandez-Velazquez *et al.*, 2005) | VIC | 4 | 60 | 14 | .880 | .878 | 0.088 | 0.577 |
| **Agaz3** | (Hoffman, 2009) | PET | 4 | 60 | 6 | .637 | .631 | 0.241 | 0.646 |
| **962-1** | Unpublished data | FAM | 5 | 60 | 4 | .524 | .571 | 0.073 | 0.577 |
| **554-6** | Unpublished data | FAM | 5 | 60 | 2 | .147 | .135 | 0.341 | 0.650 |
| **ZcwA12** | (Hoffman, Steinfartz and Wolf, 2007) | FAM | 5 | 60 | 19 | .816 | .850 | 0.094 | 0.577 |
| **PvcE** | (Coltman, Bowen and Wright, 1996) | VIC | 5 | 60 | 14 | .859 | .873 | 0.072 | 0.577 |
| **ZcwB09** | (Wolf *et al.*, 2006) | VIC | 5 | 60 | 13 | .833 | .857 | 0.781 | 0.854 |
| **Agaz10** | (Hoffman, 2009) | NED | 5 | 60 | 11 | .756 | .777 | 0.476 | 0.694 |
| **Mang44** | (Sanvito *et al.*, 2013) | PET | 5 | 60 | 6 | .677 | .716 | 0.405 | 0.683 |
| **Mang36** | (Sanvito *et al.*, 2013) | PET | 5 | 60 | 4 | .116 | .120 | 0.437 | 0.683 |
| **ZcwC11** | (Wolf *et al.*, 2006) | NED | 5 | 60 | 14 | .888 | .900 | 0.383 | 0.683 |
| **ZcwE12** | (Hoffman, Steinfartz and Wolf, 2007) | FAM | 6 | 60 | 9 | .812 | .802 | 0.863 | 0.854 |
| **Hg6.1** | (Allen *et al.*, 1995) | VIC | 6 | 60 | 13 | .865 | .860 | 0.776 | 0.854 |
| **Lc28** | (Davis *et al.*, 2002) | PET | 6 | 60 | 11 | .817 | .855 | 0.193 | 0.646 |
| **ZcwA05** | (Hoffman, Steinfartz and Wolf, 2007) | FAM | 7 | 60 | 19 | .879 | .889 | 0.290 | 0.646 |
| **101-26** | Unpublished data | HEX | 8 | 60 | 6 | .730 | .756 | 0.535 | 0.739 |
| **928-4b** | Unpublished data | HEX | 8 | 60 | 13 | .850 | .849 | 0.891 | 0.854 |
| **507-11** | Unpublished data | HEX | 8 | 60 | 4 | .425 | .462 | 0.130 | 0.591 |
| **ZcwE03** | (Wolf *et al.*, 2006) | FAM | 9 | 60 | 10 | .796 | .843 | 0.357 | 0.658 |
| **ZcwE04** | (Hoffman, Steinfartz and Wolf, 2007) | VIC | 9 | 60 | 11 | .845 | .865 | 0.826 | 0.854 |
| **ZcwDh3.6** | (Hernandez-Velazquez *et al.*, 2005) | FAM | 10 | 60 | 4 | .203 | .196 | 0.782 | 0.854 |
| **Agi01** | This study | FAM | 11 | 60 | 10 | .850 | .863 | 0.295 | 0.646 |
| **Agi02** | This study | FAM | 11 | 60 | 7 | .730 | .738 | 0.123 | 0.591 |
| **Agi03** | This study | FAM | 12 | 60 | 3 | .280 | .308 | 0.268 | 0.646 |
| **Agi05** | This study | FAM | 12 | 55 | 5 | .722 | .709 | 0.297 | 0.646 |
| **Agi06** | This study | HEX | 13 | 55 | 6 | .402 | .391 | 0.724 | 0.854 |
| **Agi07** | This study | FAM | 14 | 60 | 5 | .474 | .491 | 0.139 | 0.591 |
| **Agi04** | This study | HEX | 14 | 60 | 3 | .085 | .082 | 1.000 | 0.906 |
| **Agi08** | This study | FAM | 15 | 60 | 2 | .407 | .401 | 0.869 | 0.854 |
| **Agi09** | This study | FAM | 15 | 60 | 2 | .410 | .435 | 0.445 | 0.683 |
| **Agi10** | This study | HEX | 15 | 60 | 2 | .060 | .066 | 0.235 | 0.646 |
| **Agi11** | This study | HEX | 15 | 60 | 3 | .098 | .103 | 0.490 | 0.694 |
| **Agt10** | Hoffman et al. (2008) | VIC | 3 | 60 | 4 | .303 | .311 | 0.089 | 0.577 |
| **Agt47** | Hoffman & Nichols (2011) | PET | 3 | 60 | 3 | .530 | .511 | 0.677 | 0.850 |
