# Supplementary material for: Heterozygosity at neutral and immune loci is not associated with neonatal mortality due to microbial infection in Antarctic fur seals
Source: Ecol Evol. 2019 Jun 20;9(14):7985–96. doi: 10.1002/ece3.5317 (PMC6662382; doi:10.1002/ece3.5317)
Supplement: Supplementary file 5 [file ECE3-9-7985-s005.docx]

Supplementary Table S4. Summary of the eleven immune microsatellites developed as part of this study. “Contig” refers to the name of the contig from which the locus was mined in the transcriptome assembly of Humble *et al.* (2016). “T_a_” refers to the annealing temperature used in the PCR.

| **Locus** | **Contig** | **Gene ID** | **Primer sequence (5’–3’)** | **Repeat motif** | **T_a_ (°C)** |
| --- | --- | --- | --- | --- | --- |
| Agi01 | AgU000706 | PTPRJ_HUMAN | GGTTGGCATTTTATGTGTGTCC  TGCAGAGAGACTAAAGCCAGT | (GT)15 | 60 |
| Agi02 | AgU006175 | F6PLB9_CANLF | GGACTCCTTCAAGTTCGAATTTG  GAACACATCAGCTTGCCCTG | (GA)6 | 60 |
| Agi03 | AgU000395 | TISD_HUMAN | GCCTTGATTGTAGTCCTCAGC  GAACTAAGCTCTGCCCAAGG | (AAC)5 | 60 |
| Agi04 | AgU032202 | LEG3_CANLF | TGCTTTCCACTTTAACCCGC  CAGGTCATGATCCCAGGGTC | (AG)5 | 60 |
| Agi05 | AgU000254 | CD44_CANLF | TCCTCTTCTTCCTCCTCTTCC  AGAAGTCCCATTGGTCCTGG | (TC)13 | 55 |
| Agi06 | AgU003880 | SNAI2_MOUSE | TCTTCACTCCGGCTCCAAAT  TCCTCTCAATCTAGCTGTCAGT | (AC)12 | 55 |
| Agi07 | AgU001432 | FOXC1_HUMAN | TACATACATCCCCGTGAGCC  ATCCCTTTCCAACCCACAGT | (AT)8 | 60 |
| Agi08 | AgU005175 | ID2_PONAB | CAGAAATACACATCTCTGCCACT  TTTCAAAGGTGGAGCGTGAA | (AT)7 | 60 |
| Agi09 | AgU004366 | RAGE_BOVIN | GGGGCTGATAGATGGGGTC  GAACTGTAGCCCTGGTCCTG | (CTC)6 | 60 |
| Agi10 | AgU002020 | TF65_MOUSE | CTTTGGGTAATGTCTTCTGGGG  GAAGCTGGAGGGTAGGGATG | (TC)5 | 60 |
| Agi11 | AgU001017 | MSH6_HUMAN | TGTCTCATGAGCGTGGACTt  GCCCTATGTGTCGTCCAGTA | (TCC)5 | 60 |
